# The longitudinal association between adverse childhood experiences and persistence of psychotic-like experiences in young people: evidence from the ALSPAC birth cohort

**DOI:** 10.1017/S0033291726104383

**Published:** 2026-05-11

**Authors:** Georgie Hudson, Jessie R. Baldwin, Katharine Sykes, Craig S. Mackie, Rachel Hiller, Claire Powell, James B. Kirkbride

**Affiliations:** 1 University College London, London, UK; 2 Lived Experience Researcher

**Keywords:** ALSPAC, adverse childhood experiences, longitudinal, psychosis, trauma

## Abstract

**Background:**

Adverse childhood experiences (ACEs) are associated with increased risk of psychotic-like experiences (PLEs), but the relationship between specific adversities and the persistence of PLEs in young people remains unclear. We examined associations between distinct ACEs and the persistence of PLEs until 24 years old.

**Methods:**

Using longitudinal data from participants in the Avon Longitudinal Study of Parents and Children (ALSPAC) cohort with at least one PLE datapoint, we used group-based trajectory modeling to estimate longitudinal trajectories of PLEs from age 12–24. We examined their associations with bullying victimization, maltreatment, parental mental health problems, parental substance abuse, parental separation, and parental intimate partner violence prior to first PLE experiences.

**Results:**

Among 4,448 participants, a three-group trajectory model provided the best fit, revealing low, increasing and persistent PLE groups from ages 12–24. In fully adjusted multinomial logistic regression models, those exposed to bullying were more likely to belong to either the increasing (relative risk ratio [RRR]: 1.83, 95%CIs: 1.26–2.66) or high (RRR: 1.78, 95%CIs: 1.07–2.93) PLEs group than the low PLE group; those exposed to maltreatment were more likely to be in the increasing PLE group (RRR: 1.47, 95%CIs: 1.03–2.10). No other ACEs were associated with PLE trajectories.

**Conclusions:**

Bullying was associated with persistent PLEs up to 24 years old, independent of other forms of childhood adversity, with timing-specific effects of maltreatment on increasing symptoms emerging later in adolescence. Findings provide further evidence for the importance of prioritizing bullying and maltreatment reduction as public health targets.

## Introduction

Psychotic-like experiences (PLEs) encompass positive psychotic symptoms including hallucinations, delusions, and out-of-body experiences (Lee et al., 2016). PLEs are much more common in young people compared with adults, with period prevalence estimates suggesting that around 17% of children aged 9–12 report PLEs, falling to approximately 7.5% in adolescence and 3–4% by the mid-twenties (Kelleher et al., 2012; Sullivan et al., 2020). Consistent with this, a meta-analysis of 61 population-based cohort studies found that only around 20% of individuals reporting PLEs at baseline experienced persistence over follow-up periods of up to eight years (Linscott & van Os, 2013), indicating that most PLEs are transient.

Nevertheless, persistent PLEs are clinically important, as they are associated with an increased risk of psychotic and other psychiatric disorders. A large longitudinal study of over 6,000 young people found 22 times the odds of psychotic disorder among those with increasing PLEs over time, and over twice the odds of a non-psychotic disorder, compared with those reporting consistently low level PLEs (Zhang et al., 2019). These findings underscore the importance of identifying factors associated with the persistence, rather than mere presence, of PLEs.

One key predictor of persistent PLEs is adverse childhood experiences (ACEs), defined here as abuse, neglect, and traumatic experiences in childhood which directly affect long-term health (Goddard, 2021), and includes childhood abuse and neglect (i.e. maltreatment), through to family breakdown and bullying (Morgan & Gayer-Anderson, 2016). ACEs are strongly associated with PLEs (Mongan et al., 2019) and clinical psychosis (Turner et al., 2020). In the Avon Longitudinal Study of Parents and Children (ALSPAC), childhood trauma has been shown to increase the odds of PLEs at age 18 by almost three times when adjusting for confounders (Croft et al., 2019), and childhood victimization including bullying victimization and exposure to domestic violence increased the odds of PLEs at age 12 by approximately 1.5 times (Fisher et al., 2013).

While the associations between ACEs and psychosis are well-established, ACEs cover a broad range of experiences, and few studies have explored whether particular types of ACEs are more or less important for predicting persistent PLEs. One such study by Rammos et al. (2021) used ALSPAC data to empirically derive four longitudinal profiles of PLEs: no PLEs, transient, and persistent low- and high-frequency groups. They found that children exposed to childhood trauma between ages 0–10 were more likely to develop persistent PLEs. However, it is unclear whether the associations between ACEs and persistent PLEs might be explained by confounders that were not accounted for in the study, such as genetic susceptibility to psychosis and socioeconomic status. For example, children with higher polygenic scores for schizophrenia are at greater risk of ACEs (Baldwin et al., 2023a; Sallis et al., 2021) and developing psychosis (Perkins et al., 2020). Environmental confounding is also likely, as socioeconomic deprivation increases risk of ACEs (Walsh, McCartney, Smith, & Armour, 2019) and PLEs (Scott, Chant, Andrews, & McGrath, 2006). In addition, it is unclear if the findings would vary when defining persistent PLEs via trajectory modeling, which accounts for within-person correlation, and ensures identification of heterogeneity in the groups is data-driven.

In addition, it is also important to understand whether different ACEs might differentially affect risk of developing PLEs. A study using the E-Risk birth cohort of twins found that children exposed to physical abuse or bullying victimization were 3.16 and 1.74 times, respectively, more likely to report PLEs at age 12 than those unexposed (Arseneault et al., 2011). However, exposure to unintentional harm had a much weaker relationship with risk of PLEs at age 12, and this relationship was not consistent over time (Arseneault et al., 2011). A further study using cohort data from the Adolescent Brain Cognitive Development (ABCD) study found that bullying victimization was significantly more strongly related to PLEs in 9–11 year-olds than witnessing domestic violence, traumatic grief, or financial adversity (Karcher, Niendam, & Barch, 2020).

The present study addressed these key gaps using prospective birth cohort data from over 4,400 British children followed from birth to age 24. Specifically, we examined whether exposure to specific ACEs was associated with persistence of PLEs from childhood into adulthood, after accounting for key confounders, such as socioeconomic status and genetic susceptibility. We hypothesized that exposure to ACEs would be associated with increased likelihood of persistent PLEs, and that childhood maltreatment would confer the greatest risk.

## Methods

### Data source

We used data from the ALSPAC (Boyd et al., 2013; Fraser et al., 2013; Northstone et al., 2023). Pregnant women resident in Avon, UK with expected dates of delivery between April 1, 1991 and December 31, 1992 were invited to take part in the study. The initial sample consisted of 14,541 pregnant women and 13,988 children alive at one year. The total sample size using any data collected after the age of seven is 15,447 pregnancies, with 14,901 children alive at one year (Northstone et al., 2019). Please note that the study website contains details of all the data that are available through a fully searchable data dictionary and variable search tool (University of Bristol, 2024). Study data were collected and managed using Research Electronic Data Capture (REDCap) electronic data capture tools hosted at the University of Bristol (Harris et al., 2009). REDCap is a secure, web-based software platform designed to support data capture for research studies. Ethical approval for the study was obtained from the ALSPAC Ethics and Law Committee, the Local Research Ethics Committees, and UCL Research Ethics Committee (approval number: 27011.001). Informed consent for the use of all data collected was obtained from participants following the recommendations of the ALSPAC Ethics and Law Committee at the time. Participants can contact the study team at any time to retrospectively withdraw consent for their data to be used. Study participation is voluntary and during all data collection sweeps, information was provided on the intended use of data. Consent for biological samples was collected in accordance with the Human Tissue Act (2004).

### Analytical sample

For the present study, we applied the following inclusion criteria to define our cohort for analysis:

1. Data on the presence of PLEs at one or more timepoints,
2. Data on more than 10% of the variables measuring each ACE,
3. The first-born of twins (if applicable),
4. An intelligence quotient (IQ) of 70 or greater at age eight,
5. Measured polygenic risk scores (PRSs) for schizophrenia and depression,
6. White ethnicity, as reported by the mother at 32 weeks’ gestation. Given that a very high proportion of children in ALSPAC were from a White ethnic background (96.1% of total cohort; Boyd et al., 2013), and that PRSs available in the cohort perform less well for non-European ancestries (Martin et al., 2019), we restricted the sample to participants of White ethnicity. Previous studies have demonstrated a very high concordance rate between self-reported ethnicity and genetically-inferred ancestry, particularly for those with majority European ancestry (> 99%; Bryc et al., 2015; Shraga et al., 2017).

Given a relatively high proportion of participants with missing IQ data (n = 373; 8.4%), we only excluded participants from the cohort with clear evidence of an IQ less than 70, consistent with moderate to severe learning difficulties.

### Outcome

Data on PLEs were assessed using the PLIKSi semi-structured interview when participants were aged approximately 12, 18, and 24 years old. PLEs that interviewers rated as either ‘definite’ or ‘suspected’, and which were not attributable to hypnagogic or hypnopompic states, fever, or alcohol or drug use (Horwood et al., 2008) were included as PLEs in this study. We generated a binary measure at each timepoint of whether each child was assessed to have no PLEs or at least one suspected or definite symptom. We then modeled PLE trajectories during adolescence and young adulthood (described in detail below).

### Exposures

Our choice of ACEs to include before age 12 was guided by previous literature (Baldwin, Reuben, Newbury, & Danese, 2019; Baldwin et al., 2023a) and through consultation and discussion with our lived experience advisory group, comprising of six people with lived experience of psychosis and childhood adversity (see Supplementary Material for further information). We included six ACE domains: bullying victimization, maltreatment, parental mental health problems, parental substance abuse, parental separation, and parental intimate partner violence. Due to the low prevalence and high co-occurrence of specific types of maltreatment exposure (e.g. 2.8% for sexual abuse; Houtepen et al., 2018; Russell et al., 2019), statistical power was limited to assess the independent effects of individual subtypes of maltreatment. Therefore, we created a single measure of maltreatment, which incorporated experiences of emotional, physical, and sexual abuse and neglect, as per Baldwin et al. (2023a). Each ACE domain was assessed prospectively via questionnaires completed by the child and/or parents at multiple timepoints across multiple items from birth to age 11 years (see Supplementary Table 1 for details), all prior to the age at which PLEs were first assessed.

Each of our six ACE domains was entered into univariable and multivariable models as separate binary variables indicating whether each child had experienced the event at least once between birth and age 11.

### Covariates

We selected covariates based on our theoretical knowledge and prior evidence about potential confounders (Baldwin et al., 2023a; Boden, Fergusson, & John Horwood, 2008; Curtis, 2018; Cuttler, Mischley, & Sexton, 2016; Fisher et al., 2013; Genowska et al., 2022; Jeffers, Glantz, Byers, & Keyhani, 2021; McGrath et al., 2014; Pain et al., 2018; Perkins et al., 2020; Power et al., 2021; Sallis et al., 2021; Schürhoff et al., 2020; Scott et al., 2006; Solmi, Lewis, Zammit, & Kirkbride, 2020; Spada et al., 2018; Walsh, McCartney, Smith, & Armour, 2019). We constructed a directed acyclic graph (DAG; Supplementary Figure 1) to model the hypothesized causal pathways, and to identify relevant confounding variables. Using the DAG, the following minimal sufficient adjustment sets were identified: sex at birth (male/female), birthweight, IQ at age 8, paternal age at participant’s birth, parental depression during pregnancy, neighborhood deprivation, population density quintiles at birth, participant PRSs for schizophrenia and depression, and their ten principal components to adjust for potential population stratification, and socio-economic status, measured via parental education, income, social class, and homeownership (see Supplementary Material for full details of covariate measurement and operationalization).

### Statistical analysis

#### Missing data

We imputed missing covariate and exposure data using multiple imputation by chained equations (MICE). Twenty datasets were imputed using models with a suitable distribution including auxiliary variables (see Supplementary Material). Estimates were combined across the 20 imputed datasets using Rubin’s rules (Rubin, 1987).

Birthweight and IQ were imputed as continuous variables and passively converted into binary variables of low/normal birthweight and low/average IQ. We applied additional rules (see Houtepen et al. (2018) about the imputation process) for the imputation of ACEs. For each binary ACE domain, we coded participants as ‘exposed’ if they endorsed any of the corresponding item-level questions included in that domain; no imputation was applied in these cases. If a participant did not endorse any item in that domain and had completed 50% or more of the items for that variable, their exposure status was coded as ‘unexposed’ without imputation. If participants did not endorse any item and had completed between 10% and 50% of the items for a given ACE domain, the binary ACE variable was imputed.

#### Analysis procedure

We first summarized the demographics and characteristics of the analytical sample by estimating the median and interquartile range for continuous variables. We compared included participants with and without missing data using the *Chi*-squared test for categorical data and the Mann–Whitney U test for continuous data.

Longitudinal latent trajectory groups of PLEs at ages 12, 18, and 24 were identified using group-based trajectory modeling (GBTM) using the Stata package *traj* (Jones & Nagin, 2013). We selected the optimum number of trajectory groups and their shape based on fit statistics, considering that a lower Bayesian Information Criterion (BIC), an entropy closer to one, and an average posterior probability (AvePP) exceeding 0.7 in each group provided indication of better model fit (see Supplementary Material). We fitted the groups for participants who had data on the presence of PLEs at all three timepoints, and applied the most likely trajectory group to the remaining participants using the *outofsample* subcommand. Participants were assigned to each trajectory using the maximum posterior probability assignment rule.

We then fitted appropriate regression models (logistic regression in the event of two trajectories, or multinomial regression for greater than two trajectories) to examine the association between PLE trajectories and exposure to ACEs before the age of 12 years. We first fitted univariable models to estimate the unadjusted association between each of the six ACE domains (and covariates) and the probability of assignment to a given trajectory group (Model 1). We then fitted two multivariable regression models, first adjusting for all covariates except other ACE types (Model 2), and then fully adjusting for all covariates including other ACE types (Model 3). Analyses were conducted using Stata version 18.5 (StataCorp, 2024).

### Sensitivity analyses

As a sensitivity analysis, we compared our main results to those estimated in a sample restricted to participants with PLE data at all three timepoints. We also compared our main results with those generated in a complete case dataset.

## Results

### Missing data

**Figure 1.**
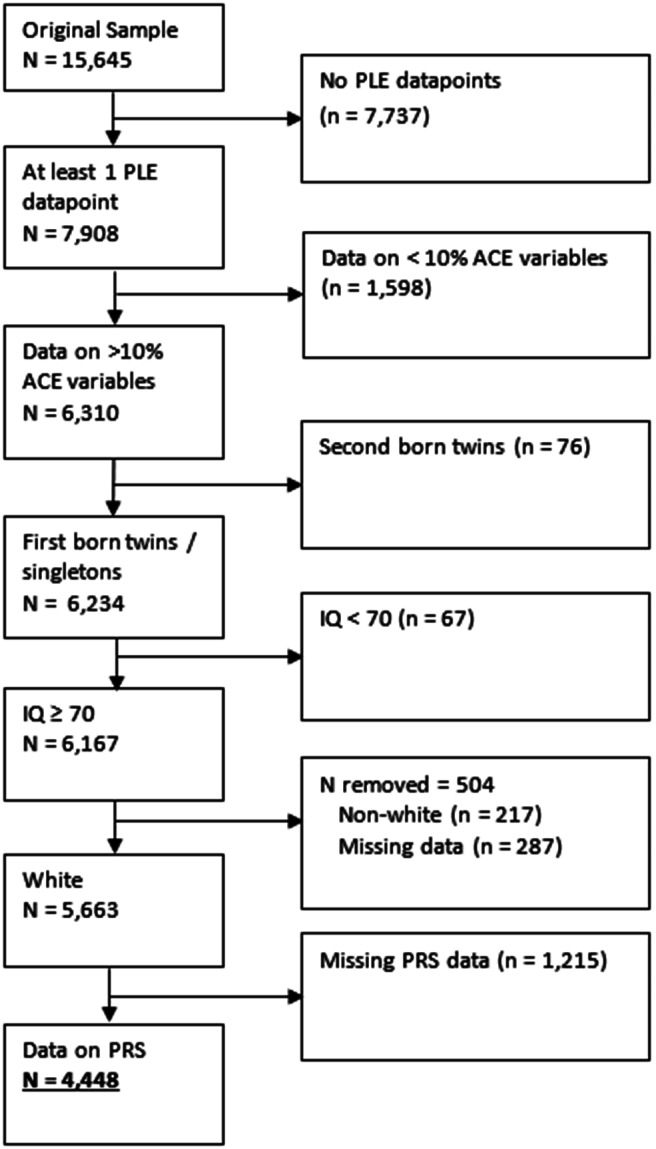
Flowchart of applying inclusion criteria to the sample, and reasons for exclusion.

In this study, 4,448 participants met inclusion criteria (Figure 1), of whom 1,007 (23%) had complete data (i.e. data on ACEs, PLEs at all three points, and all confounders; Supplementary Table 2). Those with missing covariate (n = 1871; 42%), exposure (n = 914; 21%), or some outcome (n = 2687; 60%) data were more likely to have: experienced maltreatment; been exposed to parental intimate partner violence; parents with mental health problems, drug abuse, and divorce/separation; be male; a younger father; a lower IQ; lower family income; lower maternal and paternal qualifications; lower maternal social class; their mother not owning her own home; mother or father depressed during pregnancy; higher neighborhood deprivation; and greater genetic susceptibility to both depression and schizophrenia (all p ≤ .01). Those with missing data did not differ in experiences of bullying (χ^2^(1) = 2.17, p = .14), or reporting suspected/definite PLEs at ages 12, 18, or 24 (all p > .17).

### Group-based trajectory modeling

Our GBTM of PLE symptoms indicated that a three-group linear trajectory model fitted the data best according to both BIC and entropy (Table 1) and other fit statistics (Supplementary Table 3). Entropy for the chosen model was slightly lower than desired (0.72 vs 0.80), however, this model outperformed all others considered on this metric (Supplementary Table 3). Further, there was also evidence of good correspondence between the proportion of participants assigned to a group and the estimated probability of group membership (group 1: 7% vs 11%; group 2: 90% vs 83%; group 3: 3% vs 5%), with the posterior probability having tight 95% CIs (group 1: 0.11–0.12; group 2: 0.82–0.85; group 3: 0.05–0.06). The AvePP values of group membership for participants in each group exceeded 0.7 as looked-for (group 1: 0.79; group 2: 0.92; group 3: 0.83). Although the odds of correct classification for group two did not exceed the threshold of five (2.31), this criterion was substantially exceeded for groups one and three (29 and 89, respectively).

**Table 1.** BIC and entropy values for each GBTM model

Number of groups	Trajectory shape^a^	BIC (N = 1761)^b^	Entropy
2	33	−1505.55	0.655
3	333	−1539.15	0.362
4	3333	−1557.83	0.338
5	33333	−1571.88	0.205
6	333333	−1590.56	0.162
7	3333333	−1609.25	0.130
8	33333333	−1627.93	0.123
2	22	−1513.00	0.651
3	222	−1527.94	0.419
4	2222	−1538.25	0.402
5	22222	−1553.19	0.198
6	222222	−1568.14	0.189
7	2222222	−1583.09	0.154
8	22222222	−1598.04	0.152
2	11	−1505.55	0.655
**3**	**111**	**−1512.09**	**0.722**
4	1111	−1523.30	0.514
5	11111	−1534.51	0.414
6	111111	−1545.72	0.283
7	1111111	−1556.93	0.221
8	11111111	−1568.14	0.187

*Note:* **Bold** denotes the final trajectory model chosen.

a Trajectory shapes: 1 = linear; 2 = quadratic; 3 = cubic.

b BIC, Bayesian information criterion (for the total number of participants).

**Figure 2.**
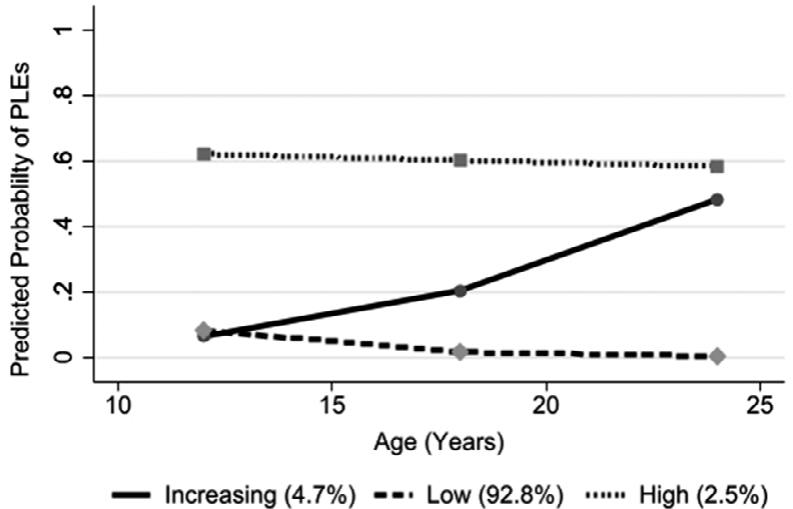
Group-based trajectory modeling (GBTM) trajectories for PLEs at ages 12, 18, and 24.

When applied to the entire sample, the chosen three trajectory group models indicated one group with low/no PLEs across the three timepoints (92.8% of the sample), one group with higher, persistent PLEs (2.5%), and one group with increasing PLEs over time (4.7%; Figure 2).

### Demographic and clinical characteristics of each trajectory group

For those assigned to the high PLE group (n = 112), all participants with data on the presence of PLEs at age 12 (n = 95 of 95; 100%) reported experiencing PLEs within the last six months. Corresponding figures at ages 18 and 24 were 80% (n = 78 of 97) and 75% (n = 60 of 80), respectively (Supplementary Table 4). For participants assigned to the increasing PLEs group, none reported PLEs at age 12, 71 (44%) at age 18, and 170 (100%) at age 24. At age 24, none of the participants assigned to the low PLEs group reported PLEs, however, a small number reported experiencing PLEs in the last six months at ages 12 and 18 (n = 375 (10%), and n = 49 (2%), respectively).

Compared with those in the low PLE group, those in the high or increasing PLE groups were more likely to have: experienced maltreatment (low: 20%; increasing: 31%; high: 31%), bullying (low: 11%; increasing: 19%; high: 19%), parental intimate partner violence (low: 22%; increasing: 30%, high: 30%), parental mental health problems (low: 25%; increasing: 32%; high: 34%), parental divorce/separation (low: 24%; increasing: 32%; high: 35%), a mother with lower education, and a mother who reported tobacco use during pregnancy (low: 17%; increasing: 23%; high: 27%) (all χ^2^ p < .05). Those in the high or increasing PLE groups had no differences in the frequency of reported parental drug abuse (χ^2^(2) = 0.67, p = .72) compared to those in the low PLE group.

Participants in the high or increasing PLE group were more likely to experience any ACEs (74% and 77%, respectively), compared with those in the low PLE group (63%; χ^2^ (2) = 21.6, *p* < .001).

### Multinomial regression modeling of the relationship between ACEs and PLE trajectories

In our univariable model, participants who experienced maltreatment were almost twice as likely to be in the increasing (relative risk ratio [RRR]: 1.74, 95% CIs: 1.27–2.38) and high (RRR: 1.78, 95% CIs: 1.18–2.70) PLE trajectory groups, compared with the low PLE trajectory group.

These effects were attenuated to a similar extent after adjustment for covariates and other ACE types, but only remained statistically significant in the increasing (RRR: 1.47, 95% CIs: 1.03–2.10) and not the high (RRR: 1.47, 95% CIs: 0.91–2.36) PLE group.

Participants who experienced bullying were also almost twice as likely to be assigned to the increasing (RRR: 1.81, 95% CIs: 1.26–2.59) and high (RRR: 1.88, 95% CIs: 1.16–3.05) PLE groups in a univariable model, compared with the low PLE trajectory group. These effects were largely unattenuated following adjustment for covariates and other ACE types (increasing PLE group RRR: 1.83, 95% CI: 1.26–2.66; high PLE group RRR: 1.78, 95% CI: 1.07–2.93).

Those exposed to parental intimate partner violence, mental health problems, or separation were also more likely to be assigned to the increasing or high PLE groups, compared with the low PLE group in univariable models (RRR range 1.43–1.56), but these effects were attenuated to the null in fully adjusted models (RRR range: 1.14–1.21). Participants exposed to parental drug abuse were no more likely to be in the high or increasing PLE groups than the low PLE group across any model. Supplementary Table 5 provides full model coefficients for all variables in the above analyses.

We observed minor differences when comparing our fully adjusted model to one which did not control for other ACE types (Model Two; Table 2). In this model, participants exposed to maltreatment remained more likely to be assigned to the high PLE group (RRR: 1.71, 95% CIs: 1.10–2.66), which did not reach statistical significance in the fully adjusted model. Additionally, participants exposed to parental intimate partner violence were more likely to be assigned to the increasing PLE group (RRR: 1.48, 95% CIs: 1.05–2.08).

**Table 2.** Univariable and multivariable multivariate regression model results for the association between ACEs and PLEs trajectory groups (20 imputations)

Characteristic	Model one^a^						Model two^b^						Model three^c^					
	Increasing PLEs			High PLEs			Increasing PLEs			High PLEs			Increasing PLEs			High PLEs		
	Relative risk ratio	95% CI		Relative risk ratio	95% CI		Relative risk ratio	95% CI		Relative risk ratio	95% CI		Relative risk ratio	95% CI		Relative risk ratio	95% CI	
		Low	High		Low	High		Low	High		Low	High		Low	High		Low	High
Maltreatment	1.74**	1.27	2.38	1.78*	1.18	2.70	1.69*	1.22	2.35	1.71*	1.10	2.66	1.47*	1.03	2.10	1.47	0.91	2.36
Bullying	1.81**	1.26	2.59	1.88*	1.16	3.05	1.90**	1.31	2.74	1.85*	1.23	3.04	1.83**	1.26	2.66	1.78*	1.07	2.93
Parental intimate partner violence	1.56*	1.13	2.15	1.61*	1.02	2.54	1.48*	1.05	2.08	1.51	0.92	2.47	1.21	0.83	1.76	1.22	0.71	2.11
Parental mental health problems	1.43*	1.04	1.96	1.66*	1.09	2.51	1.34	0.94	1.90	1.52	0.95	2.43	1.19	0.83	1.73	1.37	0.84	2.22
Parental drug abuse	1.24	0.84	1.83	0.89	0.48	1.65	1.13	0.74	1.70	0.76	0.40	1.46	0.96	0.63	1.46	0.62	0.31	1.21
Parental separation	1.47*	1.08	1.99	1.70*	1.11	2.59	1.35	0.97	1.90	1.52	0.96	2.42	1.14	0.80	1.63	1.33	0.80	2.20

a Univariable, unadjusted model.

b Adjusted for all covariates, but not other forms of ACEs.

c Adjusted for all variables in the model, including all covariates and other forms of ACEs.

* Indicates *p* < .05. ** indicates *p* < .001.

### Sensitivity analyses

We observed only minor differences between the results from analyses using the imputed and the complete case datasets (Supplementary Table 6); notably, in the complete case multivariable model, the relationship between bullying and probability of being assigned to the high PLEs trajectory group did not reach the conventional threshold for statistical significance (RRR: 1.36, 95% CIs: 0.41–4.45) unlike in the imputed dataset.

We also compared our main models with the same models restricted to participants who had complete PLE data at all three timepoints (n = 1,761 (40%); Supplementary Table 7). Again, we observed minor differences in our results; notably, in contrast to our main results the relationship between maltreatment and the high PLE trajectory group was statistically significant (RRR: 2.29, 95% CIs: 1.16–4.52) in our multivariable, fully adjusted model restricted to people with complete PLE data, while the relationship between bullying and high PLEs was not (RRR: 1.84, 95% CIs: 0.86–3.93).

## Discussion

### Principal findings

We found three trajectories of PLEs from ages 12 to 24: low, high persisting, and increasing PLEs. After controlling for important *a priori* confounders, exposure to childhood maltreatment (physical, sexual, emotional abuse, and/or neglect) was associated with approximately 1.5-fold higher risk of belonging to the increasing PLE group compared with the low group. A similar effect size was found for persistent PLEs, our smallest group, but which narrowly precluded conventional statistical significance in our fully adjusted model. Exposure to bullying was associated with almost double the risk of membership of both the increasing and high PLE groups. In contrast, no statistically significant associations were observed for parental intimate partner violence, parental mental health problems, parental substance abuse, or parental separation after full adjustment.

### Comparison with prior literature

The number and nature of PLE trajectories we identified differed from previous ALSPAC studies (Rammos et al., 2021; Thapar et al., 2012), likely reflecting methodological differences. Using latent class growth analysis to model trajectories of self-reported PLEs, Thapar et al. (2012) identified four trajectories, whereas our use of interviewer-rated PLEs may have resulted in lower endorsement rates, consistent with prior evidence that interviewer assessments are more conservative (Granö et al., 2016; Gundersen et al., 2019; Sun et al., 2022). Rammos et al. (2021) also used interviewer-rated PLEs, but classified participants using a non-latent, empirical approach into four trajectory groups: no PLEs, transient, and persistent low- and high-frequency groups, and did not adjust for confounders or examine specific ACE subtypes. Despite these differences, their finding that persistent PLEs were associated with childhood adversity aligns with our results.

In our analyses, bullying and maltreatment were more strongly associated with persistent or increasing PLEs than other ACEs (parental mental health problems, substance abuse, separation, and intimate partner violence). This fits with previous research findings that bullying is a strong predictor of persistent PLEs, with a meta-analysis of nine studies finding a weighted odds ratio of 1.76 (Trotta, Murray, & Fisher, 2015). Childhood trauma (defined broadly as physical abuse, emotional neglect, material problems in parental household and stressful life events) has also been shown to increase the odds of persistent PLEs by approximately three times (Wigman et al., 2011). Our finding that exposure to parental intimate partner violence does not predict increasing PLEs when controlling for exposure to other ACEs suggests that the relationship between parental intimate partner violence and worsening PLEs may be mediated via exposure to other ACEs. In ALSPAC, the relationship between PLEs and exposure to domestic violence has been shown to be partially mediated by anxiety, depression, locus of control, and self-esteem (Fisher et al., 2013), however, there is limited evidence investigating if other ACEs act as mediators, or whether this extends to worsening PLEs overtime – an issue that warrants further investigation.

### Strengths and limitations

Strengths of our study include a well-controlled design, employing strict inclusion and exclusion criteria, and control for important covariates informed by a DAG. We employed multiple imputation with several auxiliary variables to increase the plausibility that the data were at least missing at random, and results from this dataset were consistent with those from the complete case dataset, despite some loss of precision around estimates in the latter.

The biggest limitation of our work was attrition. Our analytical sample comprised 28% of the original ALSPAC sample, largely due to attrition over time, meaning it may not be representative of the ALSPAC cohort. To minimize data loss, we kept our inclusion criteria as broad as possible and used MICE to handle missing data. Second, our findings may not generalize to the general population given our cohort was a subset of a specific birth cohort in one part of the UK, further restricted to those of White ethnicity for our study. Third, we chose to only investigate ACEs that occurred prior to the first assessment of PLEs at age 12 to separate the temporal order of exposure to ACEs and later PLEs. This means that we did not assess possible ongoing ACEs occurring during adolescence and the impact these might have on odds of persistent PLEs. Additionally, we used binary indicators of ACEs, so we are unable to assess the impact of severity or persistence of ACEs on persistent PLEs. Finally, despite adjusting for a range of potential confounders, the associations may reflect unmeasured confounding (e.g. from genetic influences, as PRSs only capture a small proportion of heritability in psychosis and depression liability). However, previous quasi-experimental studies have suggested causal relationships between child maltreatment (Baldwin et al., 2023b) and bullying victimization (Singham et al., 2017) and psychosis.

The entropy of our chosen model was 0.72; this was slightly lower than the conventional threshold of 0.8, indicating moderate rather than sharp separation between groups, which should be kept in mind when interpreting the downstream regression analyses. This reduced entropy is likely an artifact of PLE symptoms being operationalized as binary indicators, and trajectories modeled across only three timepoints; providing less information than continuous measures across more timepoints.

To maximize the sample size and generalizability of our results, we included participants with at least one PLE datapoint (conducting trajectory analysis among those with complete PLE data, with remaining participants assigned to their most likely trajectory *post hoc*). One limitation of this approach is that those with partial PLE data may have been more likely to be misclassified to their most probable trajectory group compared with those with complete PLE data. Nonetheless, our findings were broadly similar when we restricted the analysis to participants with complete PLE data at all three timepoints, suggesting this approach may have caused minimal misclassification.

### Meaning of the findings

As we found a particularly strong association between bullying and persistent or worsening PLEs, evidence-based anti-bullying campaigns may be warranted to cut this link. A meta-analysis of 69 RCTs found that school-based anti-bullying campaigns were successful at reducing bullying and improving mental health outcomes, albeit with small effect sizes (Fraguas et al., 2021). However, none of the included studies looked at PLEs or psychosis as an outcome, with most that investigated mental health outcomes focusing on common mental disorders including depression and anxiety (Fraguas et al., 2021). A scoping review of 16 RCT and quasi-experimental studies found that all included studies reported anti-bullying interventions reduced the impact of bullying-related trauma on victims (Hikmat, Yosep, Hernawaty, & Mardhiyah, 2024). These interventions may pose a potential avenue for minimizing the risk of persistent PLEs in young people exposed to bullying, however, more work is needed to ascertain the efficacy of reducing PLEs specifically.

We found maltreatment and bullying emerged as predictors of increasing, and for bullying persistent, PLEs, and may represent particularly salient risk factors for psychosis-related outcomes. Although mechanisms remain unclear, neurobiological, cognitive, and psychological pathways linking ACEs and psychosis have been proposed. Aas et al. (2012) found that ACEs were associated with both worse cognitive performance and smaller amygdala volume in patients with first-episode psychosis. The authors suggested this volume reduction could be caused by trauma causing the central nervous system to have increased exposure to endogenous glucocorticoids, which impact neuronal growth and function. Psychological mechanisms connecting ACEs and psychosis have also been suggested, including posttraumatic avoidance, numbing, and hyperarousal (Hardy et al., 2016). These findings indicate that targeted interventions for young people exposed to trauma to support them to develop adaptive coping strategies may aid in breaking the cycle between ACEs and psychosis. A randomized clinical trial with 155 participants with a psychotic disorder and chronic PTSD found that participants who undertook prolonged exposure therapy and eye movement desensitization and reprocessing (EMDR) therapy were more likely to achieve clinical remission during treatment than patients on a waiting list, and the effects maintained at 6-month follow-up (van den Berg et al., 2015). A literature review of the mechanisms linking bullying to psychosis identified evidence for developmental, biological, and cognitive models linking these experiences (Catone, Marwaha, Lennox, & Broome, 2017). One such suggested mechanism is that childhood trauma, including bullying, can lead to a view of the self as being constantly exposed to threat and negative views of others, triggering persecutory delusions and the onset of psychosis (Catone et al., 2017; Fowler et al., 2012).

## Conclusions

We found strong evidence that maltreatment and bullying are particularly salient risk factors for experiencing persistent PLEs in the ALSPAC cohort, maintaining these associations even when controlling for other forms of childhood adversity. These findings support targeted prevention and intervention efforts focused on these specific forms of interpersonal trauma and highlight the importance of trauma-informed approaches in clinical work with young people experiencing psychotic-like symptoms. Understanding the specific pathways through which different types of childhood adversity contribute to psychotic symptom development remains a critical priority for future research and intervention development.

## Supporting information

10.1017/S0033291726104383.sm001 Hudson et al. supplementary material Hudson et al. supplementary material

## Data Availability

The informed consent obtained from ALSPAC participants does not allow the data to be made available through any third-party-maintained public repository. Supporting data are available from ALSPAC on request under the approved proposal number, B4557. Full instructions for applying for data access can be found here: http://www.bristol.ac.uk/alspac/researchers/access/. The ALSPAC study website contains details of all available data (http://www.bristol.ac.uk/alspac/researchers/our-data/).

## Supplementary material

The supplementary material for this article can be found at http://doi.org/10.1017/S0033291726104383.
