# Supplementary material for: The longitudinal association between adverse childhood experiences and persistence of psychotic-like experiences in young people: evidence from the ALSPAC birth cohort
Source: Psychol Med. 2026 May 11;56:e135. doi: 10.1017/S0033291726104383 (PMC13161824; doi:10.1017/S0033291726104383)
Supplement: Hudson et al. supplementary material [file S0033291726104383sup001.docx]

# Supplementary Materials

## Methods

**Additional Information on Exposure Measurement**

The specific ACEs to include were guided via prior literature and via consultation with our lived experience advisory group. We discussed the relevance of the 10 categories of ‘classic ACEs’: emotional abuse, physical abuse, sexual abuse, emotional neglect, physical neglect, exposure to parental intimate partner violence, parental separation or divorce, household substance abuse, and household criminality (Dube et al., 2003). We decided to include all of these, except for parental criminality. This was because the variables assessing parental criminality in ALSPAC only measured whether a parent has received a criminal conviction and did not differentiate custodial sentences and other forms. Due to this, it was felt that these variables would broadly measure minor offences not resulting in custodial sentences and that a child may be less aware of or impacted by a parent’s court conviction, compared to the other experiences. We also considered it important to assess the impact of bullying, given prior literature linking it to PLEs (Cunningham, Hoy, & Shannon, 2016; van Dam et al., 2012).

Supplementary Table 1. ACE definitions, reporter, and child’s age at assessment.

| ACE | Definition | Reporter | Child’s age at assessment |
| --- | --- | --- | --- |
| Bullying | Child was a victim of overt or relational bullying on at least a weekly basis. | Child | 8y 6m, 10y |
| Maltreatment | Child experienced any of: sexual abuse, physical abuse (parent/parent’s partner was physically cruel to child), emotional abuse (parent/parent’s partner was emotionally cruel to child), or neglect (child always feels left out of things; never understood by parents). | Child &  Parent | 8wk, 8m, 1y 6m, 1y 9m, 2y 6m, 2y 9m, 3y 6m, 3y 11m, 4y 9m, 5y 1m, 5y 9m, 6y 1m, 6y 9m, 8y 1m, 8y 9m, 9y 2m, 9y 8m, 11y 2m |
| Parental mental health problems | Parent has hurt themselves on purpose; attempted suicide; Edinburgh Postnatal Depression Scale (EPDS) score >12; diagnosed with schizophrenia. | Parent | 8w, 8m, 1y 9m, 2y 9m, 3y 11m, 5y 1m, 6y 1m, 9y 2m, 10y 2m, 11y 2m |
| Parental substance abuse | Parent smoked cannabis daily; used hard drugs (including crack, heroin, amphetamine, opiate, cocaine, methadone, meth); had a hard drug addiction; had alcoholism/drink problem; Alcohol Use Disorders Identification Test (AUDIT) score>8. | Parent | 8wk, 8m, 1y 9m, 2y 9m, 3y 11m, 5y 1m, 6y 1m, 8y 1m, 9y 2m, 11y 2m |
| Parental separation | Parents separated or divorced. | Parent | 8m, 1y 9m, 2y 9m, 3y 11m, 5y 1m, 6y 1m, 9y 2m, 11y 2m |
| Parental intimate partner violence | Parents kicked, bitten, or hit each other; physically twisted arm; throw(n) bodily; beaten each other up; choked or strangled each other; threatened each other with knife; used knife or another weapon on each other. | Parent | 8m, 1y 9m, 2y 9m, 3y 11m, 5y 1m, 6y 1m, 8y 1m, 9y 2m, 11y 2m |
| Acronyms: wk = weeks, m = months, y = years. | | | |


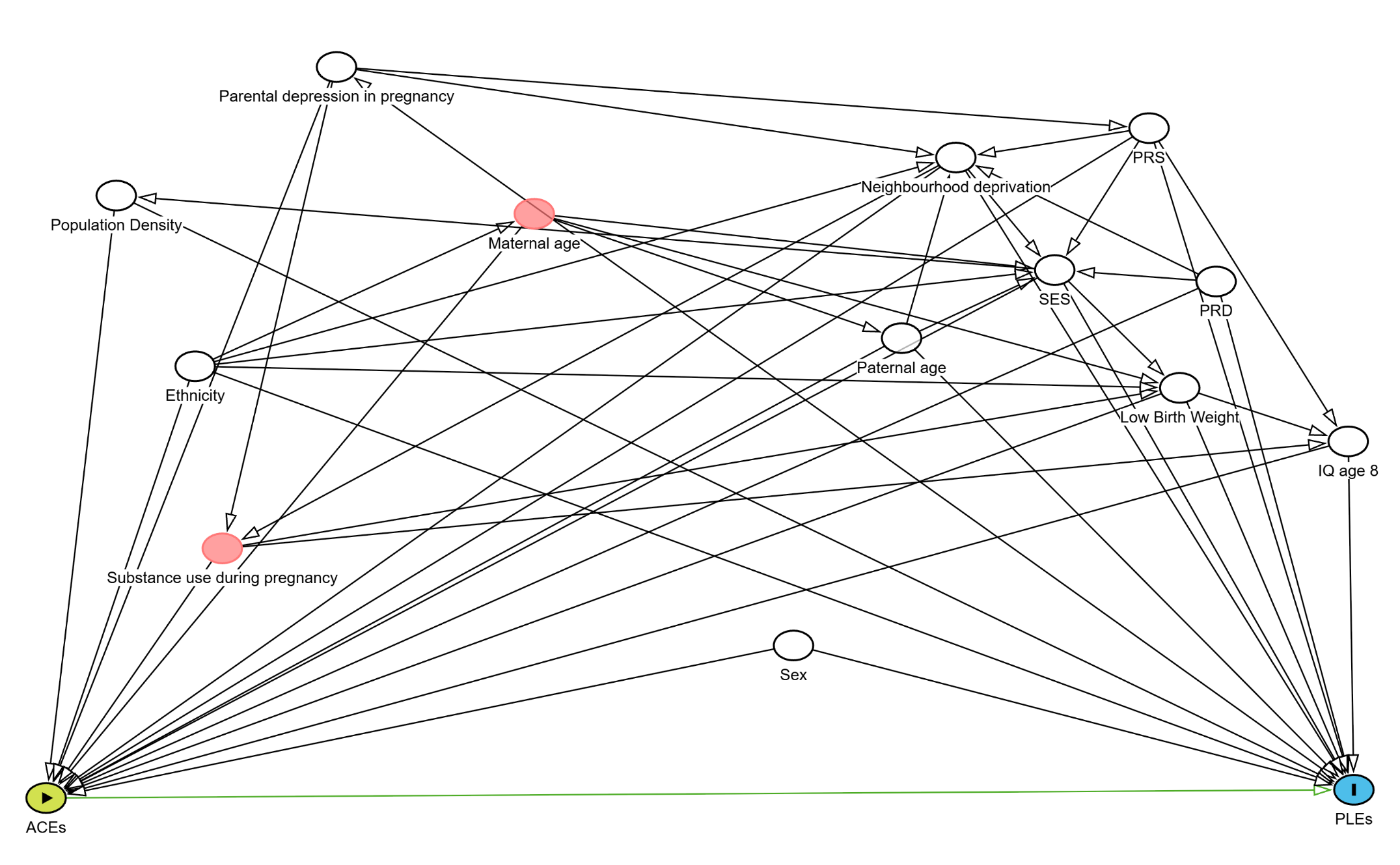


Supplementary Figure 1. Directed Acyclic Graph (DAG) constructed and used to model hypothesised causal relationships and identify confounders of the association between ACEs and PLEs.

This DAG was constructed using [www.dagitty.net](http://www.dagitty.net), and is available at <https://dagitty.net/dags.html?id=ULjcvqS6>. The green circle with triangle is the exposure (ACEs), and the blue circle with “I” is the outcome variable (PLEs). The green line shows the direct causal path between exposure and outcome. The observed confounders in the dataset impact both ACEs and PLEs, and were controlled for. The variables in pink are ancestors of both the exposure and the outcome and do not need to be controlled for..

Acronyms: ACEs: Adverse childhood experiences; PLEs: Psychotic-like experiences; SES: Socioeconomic status; PRS: Polygenic risk score for schizophrenia; PRD: Polygenic risk score for depression; IQ: Intelligence quotient.

**Additional Information on Included Covariates**

1. Ethnicity. We restricted the sample to participants of White ethnicity to control for this variable and to allow consideration of valid polygenic risk scores for schizophrenia and depression to be included as additional covariates. The proportion of children from a White ethnic background enrolled in the study is very high (96.1% of total cohort; Boyd et al., 2013), and the polygenic risk scores currently available in the ALSPAC cohort are generated based on genome-wide association studies that have been developed and optimised for those with European ancestry, making their applicability to those from different ethnic backgrounds highly limited (Martin et al., 2019). Therefore, restricting by exclusion was necessary. We defined ethnicity based on mother responses when approximately 32 weeks’ pregnant. Mothers were asked for both her and her partner’s ethnic group (White; Black Caribbean; Black African; Other Black; Indian; Pakistani; Bangladeshi; Chinese; Other). Given the small proportion of participants from a non-White ethnic group in the ALSPAC sample, we used a binary variable of the child’s ethnic background: if both parents’ ethnic groups were reported to be White, the child was defined as White; if either of the parents’ ethnic group was reported to be another ethnicity, the child’s ethnic background was defined as non-White.
2. Sex at birth (male/female).
3. Birth weight was dichotomised into low or not low birthweight. Low birthweight was defined as a birthweight of less than 2500 grams (5.5 pounds) (*UNICEF-WHO Joint Database on Low birth weight*, 2023).
4. IQ was measured via the Weschler Intelligence Scale for Children (3rd UK edition (WISC–III)) at age 8 years (Wechsler et al., 1992). As children in ALSPAC with below average IQs have previously been shown to be at the greatest risk of experiencing psychotic symptoms (Fisher et al., 2013; Horwood et al., 2008), IQ was dichotomised into: below average (an IQ of 99 or less), and average or higher (IQ of 100 or more).
5. Paternal age at time of delivery of the baby.
6. Socioeconomic status was measured via four variables:
   1. Highest parental qualification of the participant’s mother and her partner (CSE/no qualifications, vocational, O-Level, A-Level, degree level or above). Both the mother’s and her partner’s highest qualification were reported by the mother at 32-weeks’ gestation.
   2. Average weekly family income, including social benefits, was reported by the mother when her child was approximately 33 months (2.75 years old). Mothers were asked to select their average weekly income on a 5-point Likert scale: (1) Less than £100; (2) £100-£199; (3) £200-£399; (4) £300-£399; (5) More than £400.
   3. Social class of the mother and her partner were determined by the mother’s responses to questions about their jobs at 32-weeks’ gestation. To determine their social classes, the mother was asked for her and her partner’s job titles; whether they were a foreman, manager, supervisor, leading hand, or self-employed; and the type of industry they worked in. This information was then used to determine both the mother’s and her partner’s social classes using occupational codes created by the Office of Population Censuses & Surveys, using data from the 1991 census (*Standard Occupational Classification*, 1991). These social classes were: (1) unskilled; (2) semi-skilled; (3) skilled manual; (4) skilled non-manual; (5) managerial/technical; (6) professional. Also included was a separate category for those in the armed forces.
   4. Maternal home ownership was determined via a questionnaire completed by the mother as soon as she enrolled in the study, on average at 8-weeks’ gestation. The mother was asked the ownership status of her home, with the following options: (1) owned – mortgaged; (2) owned – with no mortgage to pay; (3) rented from council; (4) rented from private landlord – furnished; (5) rented from private landlord – unfurnished; (6) rented from housing association; (7) other. I subsequently recoded this data into a binary variable of whether the mother owned her home (with or without a mortgage) or did not own her home (any form of rental, or other status).
7. Parental depression in pregnancy was assessed using the Edinburgh Postnatal Depression Scale (EPDS) for the mother and her partner at 18 weeks’ gestation, and again for the mother at 32 weeks’ gestation. As for assessing ACEs, I created a binary variable of whether any of these three datapoints met or exceeded the clinical cut-off of a EPDS score of 13 or more, indicating major depression (Levis et al., 2020).
8. Neighbourhood deprivation at the point of the child’s birth was based on the Index of Multiple Deprivation (Gill, 2015). This measure is composed of 38 indicators across seven domains of deprivation (income; employment; health and disability; crime; education, skills, and training; barriers to housing and services; and living environment). Participants’ quintile ranks for the overall deprivation index across all seven domains were created by ALSPAC who linked mothers’ residential address to these indicators from 1991 Census data, in order to maintain participant anonymity. Quintile ranks range from one (low deprived) to five (high deprived). The quintile ranks therefore represent the levels of neighbourhood deprivation of each mother, relative to all other neighbourhoods in England (not just compared to other neighbourhoods recruited into ALSPAC).
9. Population density at the point of the child’s birth was similarly determined by linking mothers’ residential addresses to 1991 Census data. Population density (measured in inhabitants per km^2^) values were obtained by an intersection between population density grid maps and geocodes and were estimated as the total usual resident population in each enumeration district divided by its area size. Population density values were then rounded to the nearest 1000.
10. Polygenic risk score for schizophrenia. We used standardised polygenic risk scores (PRS) for schizophrenia derived by Kwong et al. (2021), which were shared with us for the purpose of this study. Kwong et al. (2021) generated the PRSs using summary statistics from genome-wide association study (GWAS) data from the Schizophrenia Working Group of the Psychiatric Genomics Consortium (The Schizophrenia Working Group of the Psychiatric Genomics Consortium et al., 2020; Trubetskoy et al., 2022). The PRSs were created using PRSice-2 (Choi & O’Reilly, 2019). The PRS was created by weighting the effect sizes of the single-nucleotide polymorphisms (SNPs) associated with schizophrenia from the GWAS at a p-value threshold of 0.05. PRS for schizophrenia was standardised to have a mean of 0 and a standard deviation of 1; therefore, a higher PRS represents higher genetic susceptibility to schizophrenia. The ten principal components were also included in the analyses as covariates.
11. Polygenic risk score for depression. Similarly to PRS for schizophrenia, for depression we used standardised PRS for major depressive disorder derived by Kwong et al. (2021), which used summary statistics from GWAS data from the Major Depressive Disorder Working Group of the Psychiatric Genomics Consortium (Wray et al., 2018). The PRS for depression were created in the same way as for schizophrenia, as detailed in (10) above.

**Information on auxiliary variables included in multiple imputation**

1. Parental criminality. This measured whether a child’s parent had a court conviction or was convicted of an offence other than speeding.
2. Parent-child bond assessed whether the child or parent was not close to each other when growing up, or if the child never felt loved.
   1. Parent reports many conflicts in their relationship with their child
   2. Child didn’t feel close to either parent
   3. Parent didn’t feel close to child
   4. Child rarely/never felt loved when growing up
3. Physical illness of a parent. This was a self-report measure of whether the child’s mother or her partner had had cancer.
4. Financial difficulties – Defined as present if child’s mother or her partner reported it was very difficult to afford food or heating or had become homeless at any time
5. Parity
6. Gestational age
7. Maternal BMI, based on self-reported pre-pregnancy weight

**Additional information on group-based trajectory modelling**

To determine the optimal number of trajectory groups, we started by modelling two groups with cubic polynomial functions. We then incrementally increased the number of groups by one up until eight groups were being modelled, assessing the Bayesian Information Criterion (BIC), with lower scores indicating a better-fitting model (Raftery, 1995).

However, assessing the best fitting model using only the BIC may not provide sufficient information, so in addition, to determine the optimum model we also took into account seven other factors, as suggested in previous studies (Diop et al., 2024; Logeswaran et al., 2023; Nagin & Odgers, 2010).

1. Average posterior probability (AvePP) values of group membership for participants in each group should be greater than 0.7.
2. Close correspondence between the proportion of participants assigned to a group and the estimated probability of group membership.
3. Relatively tight 95% confidence intervals around the posterior probability of group membership.
4. Higher entropy indicating better classification accuracy, ideally exceeding 0.8, demonstrating less classification uncertainty (Herle et al., 2020; Nylund-Gibson & Choi, 2018).
5. Odds of correct classification according to the posterior probabilities of group membership for each group to exceed 5.
6. Each group to have a sufficient sample size.
7. A preference for a simpler model.

Once the optimum number of groups had been established, we then varied the order of polynomial functions for each trajectory group from cubic to linear and assessed the different shape models based on the seven criteria above to identify the optimum final model. Each participant was then classified to a trajectory group. We visualised these trajectory groups of the presence of PLEs at the three timepoints using the *trajplot* Stata function (Jones & Nagin, 2013).

## Results

*Supplementary Table 2. Distribution of participant demographics and summary statistics, by complete data status.*

|  | **Missing Data** | **Complete Data** | **Statistic** | **p-value** |
| --- | --- | --- | --- | --- |
|  | **N=3,441 (77.4%)** | **N=1,007 (22.6%)** |  |  |
| **Experienced PLEs at age 12?, No. (%)** |  |  | χ^2^ (1) = 0.05 | 0.82 |
| None | 2,823 (82.0%) | 896 (89.0%) |  |  |
| Suspected or Definite | 359 (10.4%) | 111 (11.0%) |  |  |
| Missing | 259 (7.5%) | 0 (0.0%) |  |  |
| **Experienced PLEs at age 18?, No. (%)** |  |  | χ^2^ (1) = 0.64 | 0.43 |
| None | 1,742 (50.6%) | 943 (93.6%) |  |  |
| Suspected or Definite | 134 (3.9%) | 64 (6.4%) |  |  |
| Missing | 1,565 (45.5%) | 0 (0.0%) |  |  |
| **Experienced PLEs at age 24?, No. (%)** |  |  | χ^2^ (1) = 1.90 | 0.17 |
| None | 1,164 (33.8%) | 917 (91.1%) |  |  |
| Suspected or Definite | 139 (4.0%) | 90 (8.9%) |  |  |
| Missing | 2,138 (62.1%) | 0 (0.0%) |  |  |
| **Child ever maltreated? No. (%)** |  |  | χ^2^ (1) = 6.65 | 0.01 |
| No | 2,395 (69.6%) | 825 (81.9%) |  |  |
| Yes | 671 (19.5%) | 182 (18.1%) |  |  |
| Missing | 375 (10.9%) | 0 (0.0%) |  |  |
| **Child ever bullied?, No. (%)** |  |  | χ^2^ (1) = 2.17 | 0.14 |
| No | 3,014 (87.6%) | 902 (89.6%) |  |  |
| Yes | 416 (12.1%) | 105 (10.4%) |  |  |
| Missing | 11 (0.3%) | 0 (0.0%) |  |  |
| **Child ever exposed to parental intimate partner violence?, No. (%)** |  |  | χ^2^ (1) = 6.70 | 0.01 |
| No | 2,076 (60.3%) | 812 (80.6%) |  |  |
| Yes | 632 (18.4%) | 195 (19.4%) |  |  |
| Missing | 733 (21.3%) | 0 (0.0%) |  |  |
| **Parents ever had mental health problems?, No. (%)** |  |  | χ^2^ (1) = 15.29 | <.001 |
| No | 2,277 (66.2%) | 798 (79.2%) |  |  |
| Yes | 839 (24.4%) | 209 (20.8%) |  |  |
| Missing | 325 (9.4%) | 0 (0.0%) |  |  |
| **Parents ever abused drugs?, No. (%)** |  |  | χ^2^ (1) = 12.92 | <.001 |
| No | 2,574 (74.8%) | 904 (89.8%) |  |  |
| Yes | 444 (12.9%) | 103 (10.2%) |  |  |
| Missing | 423 (12.3%) | 0 (0.0%) |  |  |
| **Parents ever divorced or separated?, No. (%)** |  |  | χ^2^ (1) = 44.67 | <.001 |
| No | 2,209 (64.2%) | 839 (83.3%) |  |  |
| Yes | 823 (23.9%) | 168 (16.7%) |  |  |
| Missing | 409 (11.9%) | 0 (0.0%) |  |  |
| **Participant assigned sex at birth, No. (%)** |  |  | χ^2^ (1) = 36.33 | <.001 |
| Male | 1,745 (50.7%) | 402 (39.9%) |  |  |
| Female | 1,696 (49.3%) | 605 (60.1%) |  |  |
| **IQ Age 8, median (IQR)** | 105 (94-117) | 111 (101-122) | Z =-10.46 | <0.001 |
| **Mother's partner's age at child's birth, median (IQR)** | 31 (28-35) | 31 (29-35) | Z = -3.16 | 0.002 |
| **Mother's highest qualification, No. (%)** |  |  | χ^2^ (4) = 118.75 | <.001 |
| CSE | 384 (11.2%) | 54 (5.4%) |  |  |
| Vocational | 296 (8.6%) | 44 (4.4%) |  |  |
| O level | 1,235 (35.9%) | 309 (30.7%) |  |  |
| A level | 962 (28.0%) | 310 (30.8%) |  |  |
| Degree | 555 (16.1%) | 290 (28.8%) |  |  |
| Missing | 9 (0.3%) | 0 (0.0%) |  |  |
| **Partner's highest qualification, No. (%)** |  |  | χ^2^ (4) = 89.18 | <.001 |
| CSE | 595 (17.3%) | 92 (9.1%) |  |  |
| Vocational | 256 (7.4%) | 62 (6.2%) |  |  |
| O level | 762 (22.1%) | 198 (19.7%) |  |  |
| A level | 980 (28.5%) | 301 (29.9%) |  |  |
| Degree | 761 (22.1%) | 354 (35.2%) |  |  |
| Missing | 87 (2.5%) | 0 (0.0%) |  |  |
| **Family income per week (£), No. (%)** |  |  | χ^2^ (4) = 61.58 | <.001 |
| <100 | 133 (3.9%) | 19 (1.9%) |  |  |
| 100 - 199 | 439 (12.8%) | 95 (9.4%) |  |  |
| 200 - 299 | 840 (24.4%) | 264 (26.2%) |  |  |
| 300 - 399 | 655 (19.0%) | 263 (26.1%) |  |  |
| >400 | 769 (22.3%) | 366 (36.3%) |  |  |
| Missing | 605 (17.6%) | 0 (0.0%) |  |  |
| **Mother's social class, No. (%)** |  |  | χ^2^ (5) = 39.87 | <.001 |
| I | 201 (5.8%) | 110 (10.9%) |  |  |
| II | 1,072 (31.2%) | 421 (41.8%) |  |  |
| III (non-manual) | 1,191 (34.6%) | 375 (37.2%) |  |  |
| III (manual) | 200 (5.8%) | 40 (4.0%) |  |  |
| IV | 219 (6.4%) | 51 (5.1%) |  |  |
| V | 36 (1.0%) | 10 (1.0%) |  |  |
| Missing | 522 (15.2%) | 0 (0.0%) |  |  |
| **Does the mother own her own home (mortgaged or outright)?, No. (%)** |  |  | χ^2^ (1) = 26.94 | <.001 |
| No | 491 (14.3%) | 83 (8.2%) |  |  |
| Yes | 2,889 (84.0%) | 924 (91.8%) |  |  |
| Missing | 61 (1.8%) | 0 (0.0%) |  |  |
| **Neighbourhood deprivation quintile at child's birth, No. (%)** |  |  | χ^2^ (4) = 40.72 | <0.001 |
| 1 | 579 (16.8%) | 270 (26.8%) |  |  |
| 2 | 580 (16.9%) | 227 (22.5%) |  |  |
| 3 | 707 (20.5%) | 234 (23.2%) |  |  |
| 4 | 558 (16.2%) | 161 (16.0%) |  |  |
| 5 | 508 (14.8%) | 115 (11.4%) |  |  |
| Missing | 509 (14.8%) | 0 (0.0%) |  |  |
| **Population density quintile at child's birth (inhab/km2), No. (%)** |  |  | χ^2^ (9) = 3.46 | .94 |
| <=1000 | 327 (9.5%) | 115 (11.4%) |  |  |
| 2000 | 368 (10.7%) | 138 (13.7%) |  |  |
| 3000 | 287 (8.3%) | 103 (10.2%) |  |  |
| 4000 | 583 (16.9%) | 195 (19.4%) |  |  |
| 5000 | 370 (10.8%) | 130 (12.9%) |  |  |
| 6000 | 166 (4.8%) | 60 (6.0%) |  |  |
| 7000 | 165 (4.8%) | 61 (6.1%) |  |  |
| 8000 | 180 (5.2%) | 72 (7.1%) |  |  |
| 9000 | 143 (4.2%) | 56 (5.6%) |  |  |
| >=10000 | 253 (7.4%) | 77 (7.6%) |  |  |
| Missing | 599 (17.4%) | 0 (0.0%) |  |  |
| **Mother or father depressed during pregnancy?, No. (%)** |  |  | χ^2^ (1) = 8.89 | .003 |
| No | 2,673 (77.7%) | 857 (85.1%) |  |  |
| Yes | 628 (18.3%) | 150 (14.9%) |  |  |
| Missing | 140 (4.1%) | 0 (0.0%) |  |  |
| **Polygenic risk score for depression, median (IQR)** | -0.08 (-0.73-0.65) | -0.16 (-0.81-0.54) | Z = 2.58 | 0.010 |
| **Polygenic risk score for schizophrenia, median (IQR)** | -0.03 (-0.71-0.65) | -0.12 (-0.80-0.48) | Z = 3.84 | <0.001 |
| *Abbreviations:* d.f. Degrees of freedom; IQR: interquartile range | | | | |

Supplementary Table 3. Model fit statics for two-group and three-group linear trajectory models

|  |  |  |  | **Group** | | | |
| --- | --- | --- | --- | --- | --- | --- | --- |
| **Trajectory Shape** | **BIC** | **Entropy** |  | 1 | 2 | 3 | |
| **33** | -1505.55 | 0.655 | **Estimated group probability (%)** | 87.34 | 12.66 | / | |
|  |  |  | **Posterior probability of group membership (95% CIs)** | 80.07 (78.59 – 81.55) | 19.92 (18.48 – 21.38) | / | |
|  |  |  | **AvePP** | 0.92 | >0.99 | / | |
|  |  |  | **OCC** | 2.74 | 70247650 | / | |
| **32** | Convergence not achieved | - | **Estimated group probability (%)** | - | - | / | |
|  |  |  | **Posterior probability of group membership (95% CIs)** | - | - | / | |
|  |  |  | **AvePP** | - | - | / | |
|  |  |  | **OCC** | - | - | / | |
| **31** | Convergence not achieved | - | **Estimated group probability (%)** | - | - | / | |
|  |  |  | **Posterior probability of group membership (95% CIs)** | - | - | / | |
|  |  |  | **AvePP** | - | - | / | |
|  |  |  | **OCC** | - | - | / | |
| **23** | -1516.73 | 0.651 | **Estimated group probability (%)** | 87.34 | 12.66 | / | |
|  |  |  | **Posterior probability of group membership (95% CIs)** | 0.80 (0.79 – 0.81) | 0.20 (0.19 – 0.21) | / | |
|  |  |  | **AvePP** | 0.92 | >0.99 | / | |
|  |  |  | **OCC** | 2.12 | 10932455 | / | |
| **22** | -1513.00 | 0.651 | **Estimated group probability (%)** | 87.34 | 12.66 | / | |
|  |  |  | **Posterior probability of group membership (95% CIs)** | 0.80 (0.79 – 0.81) | 0.20 (0.19 – 0.21) | / | |
|  |  |  | **AvePP** | 0.92 | >0.99 | / | |
|  |  |  | **OCC** | 2.71 | 70268 | / | |
| **2 1** | Convergence not achieved | - | **Estimated group probability (%)** | - | - | / | |
|  |  |  | **Posterior probability of group membership (95% CIs)** | - | - | / | |
|  |  |  | **AvePP** | - | - | / | |
|  |  |  | **OCC** | - | - | / | |
| **1 3** | -1513.00 | 0.651 | **Estimated group probability (%)** | 87.34 | 12.66 | / | |
|  |  |  | **Posterior probability of group membership (95% CIs)** | 0.80 (0.79 – 0.81) | 0.20 (0.19 – 0.21) | / | |
|  |  |  | **AvePP** | 0.92 | >0.99 | / | |
|  |  |  | **OCC** | 2.71 | 25933673 | / | |
| **1 2** | -1509.26 | 0.651 | **Estimated group probability (%)** | 87.34 | 12.66 | / | |
|  |  |  | **Posterior probability of group membership (95% CIs)** | 0.80 (0.79 – 0.81) | 0.20 (0.19 – 0.21) | / | |
|  |  |  | **AvePP** | 0.92 | >0.99 | / | |
|  |  |  | **OCC** | 2.71 | 17794596 | / | |
| **1 1** | -1505.55 | 0.655 | **Estimated group probability (%)** | 87.34 | 12.66 | / | |
|  |  |  | **Posterior probability of group membership (95% CIs)** | 0.80 (0.79 - 0.81) | 0.20 (0.18 – 0.21) | / | |
|  |  |  | **AvePP** | 0.92 | >0.99 | / | |
|  |  |  | **OCC** | 2.74 | 70247650 | / | |
| **1 1 1** | **-1512.09** | **0.722** | **Estimated group probability (%)** | **6.53** | **90.12** | **3.35** | |
|  |  |  | **Posterior probability of group membership (95% CIs)** | **0.11 (0.11 – 0.12)** | **0.83 (0.82 – 0.85)** | **0.054 (0.046 – 0.062)** | |
|  |  |  | **AvePP** | **0.79** | **0.92** | **0.83** | |
|  |  |  | **OCC** | **28.65** | **2.31** | **88.52** | |
| Note: **Bold** denotes the final trajectory model chosen.  Abbreviations: AvePP = average posterior probability; OCC = odds of correct classification.  ^1^Trajectory shapes: 1= linear; 2 = quadratic; 3 = cubic. | | | | | | |  |

*Supplementary Table 4. Distribution of participant demographics and summary statistics, by PLE trajectory group.*

|  | **Low PLEs**  **N=4,126**  **(92.8%)** | **Increasing PLEs** | **High PLEs**  **N=112**  **(2.5%)** | **Statistic (df)** | **p-value** |
| --- | --- | --- | --- | --- | --- |
|  |  | **N=210**  **(4.7%)** |  |  |  |
| Experienced PLEs at age 12?, No. (%) |  |  |  | 𝜒2(4) =732.72 | <0.001 |
| None | 3,527 (85.5%) | 192 (91.4%) | 0 (0.0%) |  |  |
| Suspected or Definite | 375 (9.1%) | 0 (0.0%) | 95 (84.8%) |  |  |
| Missing | 224 (5.4%) | 18 (8.6%) | 17 (15.2%) |  |  |
| Experienced PLEs at age 18?, No. (%) |  |  |  | 𝜒2(4) =1648.47 | <0.001 |
| None | 2,575 (62.4%) | 91 (43.3%) | 19 (17.0%) |  |  |
| Suspected or Definite | 49 (1.2%) | 71 (33.8%) | 78 (69.6%) |  |  |
| Missing | 1,502 (36.4%) | 48 (22.9%) | 15 (13.4%) |  |  |
| Experienced PLEs at age 24?, No. (%) |  |  |  | 𝜒2(4) =3222.62 | <0.001 |
| None | 2,060 (49.9%) | 0 (0.0%) | 21 (18.8%) |  |  |
| Suspected or Definite | 0 (0.0%) | 170 (81.0%) | 59 (52.7%) |  |  |
| Missing | 2,066 (50.1%) | 40 (19.0%) | 32 (28.6%) |  |  |
| Participant assigned sex at birth, No. (%) |  |  |  | 𝜒2(4) = 19.18 | <0.001 |
| Male | 2,007 (48.6%) | 100 (47.6%) | 40 (35.7%) |  |  |
| Female | 2,119 (51.4%) | 110 (52.4%) | 72 (64.3%) |  |  |
| Child ever maltreated?, No. (%) |  |  |  |  |  |
| No | 3,017 (73.1%) | 133 (63.3%) | 70 (62.5%) | 𝜒2(4) = 18.54 | <0.001 |
| Yes | 762 (18.5%) | 59 (28.1%) | 32 (28.6%) |  |  |
| Missing | 347 (8.4%) | 18 (8.6%) | 10 (8.9%) |  |  |
| Child ever bullied?, No. (%) |  |  |  |  |  |
| No | 3,655 (88.6%) | 171 (81.4%) | 90 (80.4%) | 𝜒2(4) = 10.41 | 0.034 |
| Yes | 461 (11.2%) | 39 (18.6%) | 21 (18.8%) |  |  |
| Missing | 10 (0.2%) | 0 (0.0%) | <5 (<10%) |  |  |
| Child ever exposed to parental intimate partner violence?, No. (%) |  |  |  |  |  |
| No | 2,703 (65.5%) | 122 (58.1%) | 63 (56.2%) | 𝜒2(4) = 9.33 | 0.053 |
| Yes | 748 (18.1%) | 52 (24.8%) | 27 (24.1%) |  |  |
| Missing | 675 (16.4%) | 36 (17.1%) | 22 (19.6%) |  |  |
| Parents ever had mental health problems?, No. (%) |  |  |  |  |  |
| No | 2,876 (69.7%) | 132 (62.9%) | 67 (59.8%) | 𝜒2(4) = 2.79 | 0.59 |
| Yes | 952 (23.1%) | 61 (29.0%) | 35 (31.2%) |  |  |
| Missing | 298 (7.2%) | 17 (8.1%) | 10 (8.9%) |  |  |
| Parents ever abused drugs?, No. (%) |  |  |  |  |  |
| No | 3,233 (78.4%) | 156 (74.3%) | 89 (79.5%) | 𝜒2(4) = 13.59 | 0.009 |
| Yes | 507 (12.3%) | 28 (13.3%) | 12 (10.7%) |  |  |
| Missing | 386 (9.4%) | 26 (12.4%) | 11 (9.8%) |  |  |
| Parents ever divorced or separated?, No. (%) |  |  |  |  |  |
| No | 2,848 (69.0%) | 134 (63.8%) | 66 (58.9%) | 𝜒2(2) = 7.34 | 0.026 |
| Yes | 894 (21.7%) | 62 (29.5%) | 35 (31.2%) |  |  |
| Missing | 384 (9.3%) | 14 (6.7%) | 11 (9.8%) |  |  |
| IQ Age 8, median (IQR) | 106 (96-118) | 107 (98-118) | 106 (94-115) | 𝜒2(2) = 2.25 | 0.33 |
| Mother's partner's age at child's birth, median (IQR) | 31 (28-35) | 31 (28-34) | 31 (28-34) | 𝜒2(2) = 1.39 | 0.50 |
| Mother's highest qualification, No. (%) |  |  |  | 𝜒2(10) = 20.09 | 0.028 |
| CSE | 404 (9.8%) | 24 (11.4%) | 10 (8.9%) |  |  |
| Vocational | 316 (7.7%) | 19 (9.0%) | 5 (4.5%) |  |  |
| O level | 1,420 (34.4%) | 69 (32.9%) | 55 (49.1%) |  |  |
| A level | 1,203 (29.2%) | 47 (22.4%) | 22 (19.6%) |  |  |
| Degree | 774 (18.8%) | 51 (24.3%) | 20 (17.9%) |  |  |
| Missing | 9 (0.2%) | 0 (0.0%) | 0 (0.0%) |  |  |
| Partner's highest qualification, No. (%) |  |  |  | 𝜒2(10) = 11.59 | 0.31 |
| CSE | 629 (15.2%) | 40 (19.0%) | 18 (16.1%) |  |  |
| Vocational | 292 (7.1%) | 18 (8.6%) | 8 (7.1%) |  |  |
| O level | 903 (21.9%) | 33 (15.7%) | 24 (21.4%) |  |  |
| A level | 1,197 (29.0%) | 56 (26.7%) | 28 (25.0%) |  |  |
| Degree | 1,028 (24.9%) | 58 (27.6%) | 29 (25.9%) |  |  |
| Missing | 77 (1.9%) | 5 (2.4%) | 5 (4.5%) |  |  |
| Family income per week (£), No. (%) |  |  |  | 𝜒2(10) = 13.69 | 0.19 |
| <100 | 134 (3.2%) | 13 (6.2%) | 5 (4.5%) |  |  |
| 100 - 199 | 484 (11.7%) | 31 (14.8%) | 19 (17.0%) |  |  |
| 200 - 299 | 1,025 (24.8%) | 49 (23.3%) | 30 (26.8%) |  |  |
| 300 - 399 | 863 (20.9%) | 37 (17.6%) | 18 (16.1%) |  |  |
| >400 | 1,057 (25.6%) | 55 (26.2%) | 23 (20.5%) |  |  |
| Missing | 563 (13.6%) | 25 (11.9%) | 17 (15.2%) |  |  |
| Mother's social class, No. (%) |  |  |  | 𝜒2(12) = 8.83 | 0.72 |
| I | 293 (7.1%) | 11 (5.2%) | 7 (6.2%) |  |  |
| II | 1,393 (33.8%) | 66 (31.4%) | 34 (30.4%) |  |  |
| III (non-manual) | 1,448 (35.1%) | 81 (38.6%) | 37 (33.0%) |  |  |
| III (manual) | 220 (5.3%) | 15 (7.1%) | 5 (4.5%) |  |  |
| IV | 246 (6.0%) | 13 (6.2%) | 11 (9.8%) |  |  |
| V | 41 (1.0%) | <5 (<2%) | <5 (<2%) |  |  |
| Missing | 485 (11.8%) | 21 (10.0%) | 16 (14.3%) |  |  |
| Does the mother own her own home (mortgaged or outright)?, No. (%) |  |  |  | 𝜒2(4) = 9.55 | 0.049 |
| No | 517 (12.5%) | 35 (16.7%) | 22 (19.6%) |  |  |
| Yes | 3,553 (86.1%) | 173 (82.4%) | 87 (77.7%) |  |  |
| Missing | 56 (1.4%) | <5 (<2%) | <5 (<3%) |  |  |
| Neighbourhood deprivation quintile at child's birth (1 low, 5 high), No. (%) |  |  |  | 𝜒2(10) = 10.57 | 0.39 |
| 1 | 794 (19.2%) | 34 (16.2%) | 21 (18.8%) |  |  |
| 2 | 754 (18.3%) | 31 (14.8%) | 22 (19.6%) |  |  |
| 3 | 864 (20.9%) | 53 (25.2%) | 24 (21.4%) |  |  |
| 4 | 664 (16.1%) | 43 (20.5%) | 12 (10.7%) |  |  |
| 5 | 578 (14.0%) | 25 (11.9%) | 20 (17.9%) |  |  |
| Missing | 472 (11.4%) | 24 (11.4%) | 13 (11.6%) |  |  |
| Population density quintile at child's birth (inhab/km2), No. (%) |  |  |  | 𝜒2(20) = 9.32 | 0.98 |
| <=1000 | 408 (9.9%) | 19 (9.0%) | 15 (13.4%) |  |  |
| 2000 | 464 (11.2%) | 29 (13.8%) | 13 (11.6%) |  |  |
| 3000 | 366 (8.9%) | 16 (7.6%) | 8 (7.1%) |  |  |
| 4000 | 724 (17.5%) | 37 (17.6%) | 17 (15.2%) |  |  |
| 5000 | 467 (11.3%) | 25 (11.9%) | 8 (7.1%) |  |  |
| 6000 | 210 (5.1%) | 9 (4.3%) | 7 (6.2%) |  |  |
| 7000 | 208 (5.0%) | 10 (4.8%) | 8 (7.1%) |  |  |
| 8000 | 234 (5.7%) | 12 (5.7%) | 6 (5.4%) |  |  |
| 9000 | 183 (4.4%) | 8 (3.8%) | 8 (7.1%) |  |  |
| >=10000 | 305 (7.4%) | 17 (8.1%) | 8 (7.1%) |  |  |
| Missing | 557 (13.5%) | 28 (13.3%) | 14 (12.5%) |  |  |
| Mother or father depressed during pregnancy?, No. (%) |  |  |  | 𝜒2(4) = 6.44 | 0.17 |
| No | 3,290 (79.7%) | 160 (76.2%) | 80 (71.4%) |  |  |
| Yes | 709 (17.2%) | 43 (20.5%) | 26 (23.2%) |  |  |
| Missing | 127 (3.1%) | 7 (3.3%) | 6 (5.4%) |  |  |
| Polygenic risk score for depression, median (IQR) | -0.09 (-0.75-0.60) | -0.25 (-0.81-0.65) | 0.07 (-0.55-0.89) | 𝜒2(2) = 4.00 | 0.14 |
| Polygenic risk score for schizophrenia, median (IQR) | -0.06 (-0.74-0.62) | 0.03 (-0.59-0.65) | -0.08 (-0.75-0.56) | 𝜒2(2) = 1.00 | 0.61 |

*Acronyms:* d.f.: Degrees of freedom; IQR: interquartile range

| Supplementary Table 5. Univariable and multivariable multinomial logistic regression model results for the association between ACEs and PLEs trajectory groups (20 imputations) | | | | | | | | | | | | | | |
| --- | --- | --- | --- | --- | --- | --- | --- | --- | --- | --- | --- | --- | --- | --- |
| **Characteristic** | | **Univariable Model** | | | | | | **Multivariable Model^** | | | | | | |
|  |  | **Increasing PLEs** | | | **High PLEs** | | | **Increasing PLEs** | | | **High PLEs** | | | |
|  |  | Relative Risk Ratio | 95 % CI | | Relative Risk  Ratio | 95 % CI | | Relative Risk Ratio | 95 % CI | | Relative Risk Ratio | 95 % CI | |  |
|  |  |  | Low | High |  | High | Low |  | Low | High |  | Low | High |  |
| Maltreatment | | 1.74** | 1.27 | 2.38 | 1.78* | 1.18 | 2.70 | 1.47* | 1.03 | 2.10 | 1.47 | 0.91 | 2.36 |  |
| Bullying | | 1.81** | 1.26 | 2.59 | 1.88* | 1.16 | 3.05 | 1.83** | 1.26 | 2.66 | 1.78* | 1.07 | 2.93 |  |
| Parental intimate partner violence | | 1.56* | 1.13 | 2.15 | 1.61* | 1.02 | 2.54 | 1.21 | 0.83 | 1.76 | 1.22 | 0.71 | 2.11 |  |
| Parental mental health problems | | 1.43* | 1.04 | 1.96 | 1.66* | 1.09 | 2.51 | 1.19 | 0.83 | 1.73 | 1.37 | 0.84 | 2.22 |  |
| Parental drug abuse | | 1.24 | 0.84 | 1.83 | 0.89 | 0.48 | 1.65 | 0.96 | 0.63 | 1.46 | 0.62 | 0.31 | 1.21 |  |
| Parental separation | | 1.47* | 1.08 | 1.99 | 1.70* | 1.11 | 2.59 | 1.14 | 0.80 | 1.63 | 1.33 | 0.80 | 2.20 |  |
| Birth sex | |  |  |  |  |  |  |  |  |  |  |  |  |  |
|  | Male (ref) | 1 |  |  | 1 |  |  | 1 |  |  | 1 |  |  |  |
|  | Female | 1.04 | 0.79 | 1.38 | 1.70 | 1.15 | 2.52 | 1.09 | 0.82 | 1.44 | 1.69* | 1.13 | 2.53 |  |
| Low birthweight | | 0.65 | 0.27 | 1.61 | 0.97 | 0.35 | 2.68 | 0.62 | 0.25 | 1.54 | 1.02 | 0.36 | 2.89 |  |
| IQ | |  |  |  |  |  |  |  |  |  |  |  |  |  |
|  | Below average | 0.83 | 0.61 | 1.15 | 1.16 | 0.77 | 1.74 | 0.72 | 0.51 | 1.02 | 1.05 | 0.67 | 1.63 |  |
|  | Average or above (ref) | 1 |  |  | 1 |  |  | 1 |  |  | 1 |  |  |  |
| Paternal age | | 0.99 | 0.97 | 1.02 | 0.97 | 0.94 | 1.01 | 1.00 | 0.97 | 1.02 | 0.99 | 0.95 | 1.03 |  |
| Mother’s highest qualification | |  |  |  |  |  |  |  |  |  |  |  |  |  |
|  | CSE | 0.90 | 0.55 | 1.48 | 0.96 | 0.44 | 2.06 | 0.65 | 0.33 | 1.26 | 0.87 | 0.32 | 2.39 |  |
|  | Vocational | 0.91 | 0.53 | 1.57 | 0.61 | 0.23 | 1.64 | 0.70 | 0.36 | 1.35 | 0.63 | 0.20 | 1.98 |  |
|  | O-Level | 0.74 | 0.51 | 1.07 | 1.50 | 0.89 | 2.51 | 0.61 | 0.37 | 1.01 | 1.63 | 0.79 | 3.39 |  |
|  | A-Level | 0.59* | 0.39 | 0.89 | 0.71 | 0.38 | 1.30 | 0.52* | 0.33 | 0.42 | 0.74 | 0.36 | 1.50 |  |
|  | Degree or above (ref) | 1 |  |  | 1 |  |  | 1 |  |  | 1 |  |  |  |
| Mother’s partner’s highest qualification | |  |  |  |  |  |  |  |  |  |  |  |  |  |
|  | CSE | 1.13 | 0.75 | 1.70 | 1.05 | 0.58 | 1.90 | 1.05 | 0.60 | 1.84 | 0.61 | 0.27 | 1.34 |  |
|  | Vocational | 1.11 | 0.64 | 1.91 | 0.99 | 0.45 | 2.17 | 1.14 | 0.61 | 2.15 | 0.64 | 0.60 | 1.60 |  |
|  | O-Level | 0.66 | 0.43 | 1.02 | 0.96 | 0.56 | 1.66 | 0.67 | 0.40 | 1.13 | 0.60 | 0.30 | 1.19 |  |
|  | A-Level | 0.84 | 0.58 | 1.22 | 0.84 | 0.50 | 1.43 | 0.91 | 0.59 | 1.42 | 0.67 | 0.36 | 1.24 |  |
|  | Degree or above (ref) | 1 |  |  | 1 |  |  | 1 |  |  | 1 |  |  |  |
| Family income per week (£) | |  |  |  |  |  |  |  |  |  |  |  |  |  |
|  | <100 | 2.01* | 1.09 | 3.71 | 1.71 | 0.63 | 4.59 | 1.72 | 0.82 | 3.62 | 1.30 | 0.40 | 4.24 |  |
|  | 100 - 199 | 1.34 | 0.86 | 2.09 | 1.86* | 1.00 | 3.46 | 1.36 | 0.79 | 2.34 | 1.74 | 0.80 | 3.83 |  |
|  | 200 - 299 | 0.97 | 0.66 | 1.42 | 1.42 | 0.81 | 2.47 | 1.02 | 0.66 | 1.60 | 1.48 | 0.77 | 2.86 |  |
|  | 300 - 399 | 0.84 | 0.55 | 1.28 | 1.02 | 0.55 | 1.90 | 0.88 | 0.56 | 1.38 | 1.07 | 0.55 | 2.07 |  |
|  | >400 (ref) | 1 |  |  | 1 |  |  | 1 |  |  | 1 |  |  |  |
| Mother’s social class | |  |  |  |  |  |  |  |  |  |  |  |  |  |
|  | I (ref) | 1 |  |  | 1 |  |  | 1 |  |  | 1 |  |  |  |
|  | II | 1.21 | 0.63 | 2.32 | 1.04 | 0.45 | 2.39 | 1.57 | 0.79 | 3.14 | 0.99 | 0.39 | 2.47 |  |
|  | III (non-manual) | 1.39 | 0.73 | 2.63 | 1.12 | 0.50 | 2.54 | 1.93 | 0.92 | 4.04 | 0.91 | 0.34 | 2.46 |  |
|  | III (manual) | 1.56 | 0.70 | 3.44 | 0.98 | 0.31 | 3.06 | 1.94 | 0.79 | 4.73 | 0.73 | 0.20 | 2.67 |  |
|  | IV | 1.27 | 0.56 | 2.90 | 1.77 | 0.66 | 4.74 | 1.50 | 0.58 | 3.83 | 1.25 | 0.38 | 4.09 |  |
|  | V | 1.59 | 0.43 | 5.82 | 1.90 | 0.40 | 9.11 | 1.66 | 0.41 | 6.83 | 1.15 | 0.20 | 6.55 |  |
| Does the mother own her own home (mortgaged or outright)? | |  |  |  |  |  |  |  |  |  |  |  |  |  |
|  | No | 1.38 | 0.95 | 2.02 | 1.74 | 1.08 | 2.80 | 1.09 | 0.69 | 1.70 | 1.21 | 0.67 | 2.18 |  |
|  | Yes (ref) | 1 |  |  | 1 |  |  | 1 |  |  | 1 |  |  |  |
| Neighbourhood deprivation quintile at child's birth (1: low, 5: high) | |  |  |  |  |  |  |  |  |  |  |  |  |  |
|  | 1 (ref) | 1 |  |  | 1 |  |  | 1 |  |  | 1 |  |  |  |
|  | 2 | 0.96 | 0.59 | 1.55 | 1.13 | 0.62 | 2.05 | 0.91 | 0.55 | 1.48 | 1.00 | 0.54 | 1.86 |  |
|  | 3 | 1.38 | 0.89 | 2.12 | 1.10 | 0.61 | 2.00 | 1.32 | 0.84 | 2.08 | 0.95 | 0.51 | 1.77 |  |
|  | 4 | 1.45 | 0.92 | 2.28 | 0.77 | 0.38 | 1.58 | 1.37 | 0.84 | 2.25 | 0.60 | 0.28 | 1.28 |  |
|  | 5 | 1.02 | 0.61 | 1.70 | 1.38 | 0.74 | 2.61 | 0.85 | 0.48 | 1.52 | 0.99 | 0.48 | 2.04 |  |
| Population density quintile at child's birth (inhab/km2), | |  |  |  |  |  |  |  |  |  |  |  |  |  |
|  | <=1000 (ref) | 1 |  |  | 1 |  |  | 1 |  |  | 1 |  |  |  |
|  | 2000 | 1.23 | 0.69 | 2.19 | 0.80 | 0.38 | 1.69 | 1.20 | 0.67 | 2.17 | 0.79 | 0.37 | 1.70 |  |
|  | 3000 | 0.91 | 0.46 | 1.77 | 0.68 | 0.28 | 1.61 | 0.94 | 0.48 | 1.86 | 0.69 | 0.28 | 1.67 |  |
|  | 4000 | 1.04 | 0.60 | 1.80 | 0.71 | 0.35 | 1.43 | 1.06 | 0.61 | 1.84 | 0.71 | 0.35 | 1.46 |  |
|  | 5000 | 1.11 | 0.61 | 2.02 | 0.54 | 0.23 | 1.28 | 1.11 | 0.60 | 2.05 | 0.52 | 0.22 | 1.23 |  |
|  | 6000 | 0.89 | 0.41 | 1.98 | 0.92 | 0.37 | 2.27 | 0.91 | 0.41 | 2.04 | 0.80 | 0.32 | 2.03 |  |
|  | 7000 | 0.94 | 0.44 | 2.03 | 1.06 | 0.44 | 2.53 | 0.85 | 0.39 | 1.86 | 1.01 | 0.41 | 2.49 |  |
|  | 8000 | 1.05 | 0.51 | 2.16 | 0.71 | 0.27 | 1.87 | 0.96 | 0.46 | 2.01 | 0.73 | 0.27 | 1.96 |  |
|  | 9000 | 0.91 | 0.40 | 2.11 | 1.21 | 0.50 | 2.90 | 0.81 | 0.34 | 1.88 | 1.12 | 0.45 | 2.77 |  |
|  | >=10000 | 1.24 | 0.64 | 2.42 | 0.76 | 0.31 | 1.83 | 1.10 | 0.55 | 2.18 | 0.71 | 0.29 | 1.76 |  |
| Mother or father depressed during pregnancy | | 1.24 | 0.88 | 1.76 | 1.46 | 0.93 | 2.81 | 0.91 | 0.61 | 1.35 | 0.99 | 0.58 | 1.67 |  |
| PRS for depression | | 0.96 | 0.83 | 1.11 | 1.21 | 1.00 | 1.46 | 0.93 | 0.80 | 1.07 | 1.15 | 0.95 | 1.40 |  |
| PRS for schizophrenia | | 1.06 | 0.92 | 1.22 | 0.97 | 0.80 | 1.17 | 1.06 | 0.92 | 1.23 | 0.96 | 0.79 | 1.16 |  |
| Principal component 1 | | 4.05 | 1.79e^-08^ | 9.16e^08^ | 1.16e^-07^ | 3.05e^-19^ | 43874 | 0.48 | 1.32e^-09^ | 1.71e^08^ | 2.75e^-09^ | 4.06e^-21^ | 1869.67 |  |
| Principal component 2 | | 0.01 | 5.17e^-08^ | 2073 | 2.38e^-07^ | 3.10e^-14^ | 1.83 | 0.014 | 6.10e^-08^ | 3177 | 4.48e^-07^ | 3.28e^-14^ | 6.11 |  |
| Principal component 3 | | 0.42 | 1.66e^-06^ | 108577 | 52281 | 0.002 | 1.24e^12^ | 0.27 | 8.14e^-07^ | 9.11e^04^ | 297107 | 0.01 | 1.10e^13^ |  |
| Principal component 4 | | 5.82 | 2.62e^-05^ | 1294460 | 2.51e^-06^ | 1.78e^-13^ | 35.37 | 7.95 | 3.29E-05 | 1.92e^06^ | 1.27e^-06^ | 7.24e^-14^ | 22.38 |  |
| Principal component 5 | | 0.036 | 1.45e^-07^ | 8880 | 461.98 | 2.37e^-05^ | 8.99e^09^ | 0.043 | 1.32e^-07^ | 13911.4 | 165.55 | 5.29e^-06^ | 5.19e^09^ |  |
| Principal component 6 | | 290.25 | 0.0012 | 7.14e^07^ | 2414.57 | 0.00012 | 4.88e^10^ | 552.54 | 0.002 | 1.50e^08^ | 2478.17 | 1.12e^-04^ | 5.51e^10^ |  |
| Principal component 7 | | 16097 | 0.073 | 3.56e^09^ | 9.00 | 4.84e^-07^ | 1.68e^08^ | 2.92 | 1.04e^-05^ | 823065 | 7.61 | 2.47e^-07^ | 2.35e^08^ |  |
| Principal component 8 | | 1.93 | 7.62^-06^ | 488658 | 346.47 | 1.72e^-05^ | 6.99e^09^ | 4983.54 | 0.017 | 1.47e^09^ | 752.55 | 2.19e^-05^ | 2.59e^10^ |  |
| Principal component 9 | | 2549.48 | 0.011 | 5.95e^08^ | 26622.28 | 0.0015 | 4.87e^11^ | 9.29 | 3.67e^-05^ | 234910 | 151180 | 0.0063 | 3.62e^12^ |  |
| Principal component 10 | | 3.18 | 1.00e^-05^ | 723221 | 3.03e^-08^ | 1.52e^-15^ | 0.61 | 0.91 | 0.61 | 1.35 | 9.32e^-08^ | 2.91e^-15^ | 2.99 |  |
| * Indicates p<.05. ** indicates p<.001  *^ Adjusted for all variables in the model, including all covariates and other forms of ACEs.* | | | | | | | | | | | | | |  |

| *Supplementary Table 6. Complete case analysis multinomial logistic regression model results for the association between ACEs and PLEs trajectory groups (n=1,007)* | | | | | | | | | | | | | | |
| --- | --- | --- | --- | --- | --- | --- | --- | --- | --- | --- | --- | --- | --- | --- |
| **Characteristic** | | **Univariable Model** | | | | | | **Multivariable Model^** | | | | | | |
|  |  | **Increasing PLEs** | | | **High PLEs** | | | **Increasing PLEs** | | | **High PLEs** | | | |
|  |  | Relative Risk Ratio | 95 % CI | | Relative Risk Ratio | 95 % CI | | Relative Risk Ratio | 95 % CI | | Relative Risk Ratio | 95 % CI | |  |
|  |  |  | Low | High |  | High | Low |  | Low | High |  | Low | High |  |
| Maltreatment | | 2.30* | 1.33 | 3.98 | 1.58 | 0.70 | 3.57 | 2.54* | 1.31 | 4.93 | 1.59 | 0.59 | 4.28 |  |
| Bullying | | 2.81** | 1.52 | 5.21 | 1.32 | 0.45 | 3.84 | 2.77* | 1.39 | 5.51 | 1.36 | 0.41 | 4.45 |  |
| Intimate partner violence | | 1.14 | 0.62 | 2.10 | 1.14 | 0.49 | 2.66 | 0.94 | 0.45 | 1.96 | 1.16 | 0.43 | 3.12 |  |
| Parental mental health problems | | 1.14 | 0.63 | 2.08 | 1.24 | 0.55 | 2.80 | 1.01 | 0.48 | 2.15 | 1.44 | 0.52 | 3.97 |  |
| Parental drug abuse | | 0.53 | 0.19 | 1.50 | 0.26 | 0.03 | 1.91 | 0.35 | 0.12 | 1.08 | 0.25 | 0.03 | 2.05 |  |
| Parental separation | | 0.90 | 0.45 | 1.79 | 1.35 | 0.58 | 3.17 | 0.64 | 0.28 | 1.44 | 1.16 | 0.44 | 3.07 |  |
| Birth sex | |  |  |  |  |  |  |  |  |  |  |  |  |  |
|  | Male (ref) | 1 |  |  | 1 |  |  | 1 |  |  | 1 |  |  |  |
|  | Female | 0.64 | 0.39 | 1.06 | 0.99 | 0.49 | 2.02 | 0.73 | 0.42 | 1.27 | 0.82 | 0.37 | 1.80 |  |
| Low birthweight | | 0.34 | 0.05 | 2.54 | 3.92e^-06^ | / | / | 0.23 | 0.03 | 1.93 | 8.30e^-07^ | / | / |  |
| IQ | |  |  |  |  |  |  |  |  |  |  |  |  |  |
|  | Below average | 1.21 | 0.67 | 2.17 | 1.02 | 0.43 | 2.38 | 0.96 | 0.47 | 1.96 | 0.78 | 0.29 | 2.11 |  |
|  | Average or above (ref) | 1 |  |  | 1 |  |  | 1 |  |  | 1 |  |  |  |
| Paternal age | | 1.01 | 0.96 | 1.06 | 0.96 | 0.89 | 1.03 | 1.02 | 0.97 | 1.08 | 0.96 | 0.88 | 1.05 |  |
| Mother’s highest qualification | |  |  |  |  |  |  |  |  |  |  |  |  |  |
|  | CSE | 2.17 | 0.81 | 5.83 | 1.45 | 0.30 | 7.02 | 1.72 | 0.45 | 6.61 | 1.89 | 0.26 | 13.79 |  |
|  | Vocational | 3.33* | 1.28 | 8.65 | 1.90 | 0.39 | 9.31 | 2.90 | 0.81 | 10.36 | 2.52 | 0.33 | 19.43 |  |
|  | O-Level | 1.21 | 0.62 | 2.39 | 1.82 | 0.76 | 4.36 | 1.06 | 0.40 | 2.79 | 2.18 | 0.57 | 8.32 |  |
|  | A-Level | 0.98 | 0.49 | 1.99 | 0.70 | 0.24 | 2.03 | 1.00 | 0.43 | 2.37 | 0.77 | 0.21 | 2.85 |  |
|  | Degree or above (ref) | 1 |  |  | 1 |  |  | 1 |  |  | 1 |  |  |  |
| Mother’s partner’s highest qualification | |  |  |  |  |  |  |  |  |  |  |  |  |  |
|  | CSE | 1.20 | 0.50 | 2.90 | 1.44 | 0.45 | 4.63 | 0.52 | 0.16 | 1.68 | 0.99 | 0.20 | 4.95 |  |
|  | Vocational | 1.80 | 0.74 | 4.41 | 0.54 | 0.07 | 4.26 | 1.01 | 0.31 | 3.24 | 0.34 | 0.03 | 3.75 |  |
|  | O-Level | 0.86 | 0.41 | 1.80 | 1.47 | 0.60 | 3.62 | 0.61 | 0.24 | 1.58 | 1.04 | 0.28 | 3.85 |  |
|  | A-Level | 1.91 | 0.48 | 1.72 | 0.85 | 0.34 | 2.13 | 0.68 | 0.30 | 1.56 | 0.67 | 0.20 | 2.30 |  |
|  | Degree or above (ref) | 1 |  |  | 1 |  |  | 1 |  |  | 1 |  |  |  |
| Family income per week (£) | |  |  |  |  |  |  |  |  |  |  |  |  |  |
|  | <100 | 1.87 | 0.40 | 8.62 | 4.12e^-06^ | / | / | 2.30 | 0.36 | 14.54 | 6.67e^-07^ | / | / |  |
|  | 100 - 199 | 2.15 | 1.00 | 4.65 | 1.03 | 0.28 | 3.72 | 2.57 | 0.93 | 7.10 | 0.87 | 0.16 | 4.77 |  |
|  | 200 - 299 | 1.30 | 0.68 | 2.47 | 1.55 | 0.70 | 3.47 | 1.33 | 0.59 | 3.01 | 1.36 | 0.47 | 3.98 |  |
|  | 300- 399 | 0.84 | 0.41 | 1.71 | 0.57 | 0.20 | 1.63 | 0.76 | 0.34 | 1.67 | 0.39 | 1.12 | 1.29 |  |
|  | >400 (ref) | 1 |  |  | 1 |  |  | 1 |  |  | 1 |  |  |  |
| Mother’s social class | |  |  |  |  |  |  |  |  |  |  |  |  |  |
|  | I (ref) | 1 |  |  | 1 |  |  | 1 |  |  | 1 |  |  |  |
|  | II | 2.06 | 0.61 | 7.00 | 0.99 | 0.27 | 3.60 | 1.96 | 0.54 | 7.10 | 1.01 | 0.23 | 4.39 |  |
|  | III (non-manual) | 3.28 | 0.98 | 10.94 | 1.69 | 0.48 | 5.92 | 2.70 | 0.68 | 10.64 | 1.14 | 0.23 | 5.68 |  |
|  | III (manual) | 3.96 | 0.84 | 18.57 | 0.99 | 0.10 | 9.83 | 2.40 | 0.41 | 13.99 | 0.57 | 0.036 | 9.04 |  |
|  | IV | 2.26 | 0.44 | 11.63 | 1.51 | 0.24 | 9.33 | 1.47 | 0.22 | 9.67 | 0.82 | 0.078 | 8.61 |  |
|  | V | 8.67* | 1.26 | 59.62 | 4.11e^-06^ | / | / | 6.52 | 0.67 | 63.63 | 7.20e^-07^ | / | / |  |
| Does the mother own her own home (mortgaged or outright)? | |  |  |  |  |  |  |  |  |  |  |  |  |  |
|  | No | 1.60 | 0.74 | 3.48 | 1.16 | 0.35 | 3.90 | 0.95 | 0.35 | 2.57 | 1.05 | 0.24 | 4.58 |  |
|  | Yes (ref) | 1 |  |  | 1 |  |  | 1 |  |  | 1 |  |  |  |
| Neighbourhood deprivation quintile at child's birth (1: low deprived, 5: high deprived) | |  |  |  |  |  |  |  |  |  |  |  |  |  |
|  | 1 (ref) | 1 |  |  | 1 |  |  | 1 |  |  | 1 |  |  |  |
|  | 2 | 1.02 | 0.46 | 2.26 | 1.07 | 0.43 | 2.70 | 1.05 | 0.44 | 2.51 | 0.99 | 0.35 | 2.75 |  |
|  | 3 | 1.24 | 0.59 | 2.64 | 0.81 | 0.30 | 2.17 | 1.45 | 0.62 | 3.39 | 0.76 | 0.25 | 2.27 |  |
|  | 4 | 1.99 | 0.95 | 4.21 | 0.70 | 0.21 | 2.27 | 2.17 | 0.92 | 5.11 | 0.54 | 0.14 | 2.02 |  |
|  | 5 | 1.54 | 0.64 | 3.66 | 0.72 | 0.19 | 2.66 | 1.37 | 0.48 | 2.91 | 0.52 | 0.11 | 2.47 |  |
| Population density quintile at child's birth (inhab/km2), | |  |  |  |  |  |  |  |  |  |  |  |  |  |
|  | <=1000 (ref) | 1 |  |  | 1 |  |  | 1 |  |  | 1 |  |  |  |
|  | 2000 | 1.19 | 0.44 | 3.23 | 0.66 | 0.17 | 2.54 | 1.16 | 0.39 | 3.49 | 0.53 | 0.12 | 2.28 |  |
|  | 3000 | 1.08 | 0.37 | 3.21 | 0.22 | 0.02 | 1.89 | 1.17 | 0.35 | 3.85 | 0.18 | 0.019 | 2.65 |  |
|  | 4000 | 1.17 | 0.46 | 2.99 | 0.59 | 0.17 | 2.61 | 1.08 | 0.39 | 3.02 | 0.56 | 0.14 | 2.19 |  |
|  | 5000 | 1.00 | 0.35 | 2.85 | 0.70 | 0.18 | 2.67 | 1.00 | 0.32 | 3.15 | 0.55 | 0.12 | 2.48 |  |
|  | 6000 | 0.82 | 0.21 | 3.35 | 1.55 | 0.40 | 6.03 | 0.86 | 0.19 | 3.95 | 1.22 | 0.26 | 5.79 |  |
|  | 7000 | 1.36 | 0.41 | 4.50 | 0.76 | 0.14 | 4.06 | 1.16 | 0.31 | 4.36 | 0.71 | 0.12 | 4.27 |  |
|  | 8000 | 0.43 | 0.09 | 2.15 | 0.61 | 0.11 | 3.21 | 0.43 | 0.78 | 2.42 | 0.50 | 0.08 | 3.01 |  |
|  | 9000 | 0.87 | 0.21 | 3.49 | 0.81 | 0.15 | 4.31 | 0.92 | 0.20 | 4.24 | 0.90 | 0.15 | 5.46 |  |
|  | >=10000 | 1.56 | 0.52 | 4.65 | 1.25 | 0.32 | 4.82 | 1.15 | 0.34 | 3.90 | 1.34 | 0.30 | 6.10 |  |
| Mother or father depressed during pregnancy | | 1.29 | 0.67 | 2.49 | 1.04 | 0.39 | 2.74 | 0.96 | 0.42 | 2.19 | 0.84 | 0.24 | 2.91 |  |
| PRS for depression | | 0.98 | 0.76 | 1.26 | 1.21 | 0.85 | 1.73 | 1.04 | 0.79 | 1.37 | 1.27 | 0.85 | 1.91 |  |
| PRS for schizophrenia | | 0.99 | 0.76 | 1.28 | 1.09 | 0.76 | 1.56 | 0.91 | 0.68 | 1.23 | 1.09 | 0.72 | 1.65 |  |
| Principal component 1 | | 8.65e^-11^ | 4.08e^-27^ | 1834737 | 1.20e^-10^ | 2.43e^-33^ | 5.93e^12^ | 9.40e^-12^ | 1.17e^-29^ | 7541656 | 2.66e^-16^ | 1.71e^-41^ | 4.14e^09^ |  |
| Principal component 2 | | 0.005 | 4.23e^-13^ | 4.23e^-13^ | 1.04e^-06^ | 2.56e^-20^ | 4.26e^07^ | 0.31 | 1.87e^-12^ | 5.07e^10^ | 0.002 | 2.12e^-18^ | 1.31e^12^ |  |
| Principal component 3 | | 3.64e^-07^ | 9.30^e-17^ | 1425 | 789 | 1.72e^-11^ | 3.61e^16^ | 6.00e^-07^ | 1.65e^-17^ | 21797 | 206 | 2.86e^-13^ | 1.48e^17^ |  |
| Principal component 4 | | 1.35e^07^ | 0.001 | 1.69e^17^ | 0.0009 | 1.99e^-17^ | 4.07e^10^ | 340129 | 2.19e^-06^ | 5.29e^16^ | 0.00005 | 1.03e^-19^ | 2.08e^10^ |  |
| Principal component 5 | | 0.057 | 9.45e^-12^ | 3.39e^08^ | 330 | 8.52e^-12^ | 1.28e^16^ | 0.11 | 1.90e^-12^ | 6.85e^09^ | 2114806 | 7.84e^-09^ | 5.70e^20^ |  |
| Principal component 6 | | 0.0033 | 6.33e^-13^ | 1.77e^07^ | 393 | 1.14e^-11^ | 1.35e^16^ | <0.0001 | 1.44e^-16^ | 750105 | 168074 | 2.41e^-10^ | 1.17e^20^ |  |
| Principal component 7 | | 8524552 | 0.0026 | 2.76e^16^ | 4213 | 2.31e^-10^ | 7.68e^16^ | 1.78e^09^ | 0.048 | 6.57e^19^ | 398 | 5.13e^-12^ | 3.08e^16^ |  |
| Principal component 8 | | 3.83e^12^ | 1035 | 1.42e^22^ | 296912 | 1.73e^-08^ | 5.08e^18^ | 146545 | 3.35e^-06^ | 6.41e^15^ | 17836 | 2.60e^-11^ | 1.22e^19^ |  |
| Principal component 9 | | 4001 | 7.38e-^07^ | 2.17e^13^ | 4.79e^18^ | 208511 | 1.10e^32^ | 1.05e^-08^ | 2.02e^-19^ | 544 | 1.98e^20^ | 333733 | 1.18e^35^ |  |
| Principal component 10 | | 8.10e^-08^ | 1.73e^-17^ | 379 | 297277 | 2.04e^-08^ | 4.33e^18^ | 0.008 | 0.0006 | 0.10 | 0.16 | 0.004 | 5.85 |  |
| * Indicates p<.05. ** indicates p<.001  *^ Adjusted for all variables in the model, including all covariates and other forms of ACEs.* | | | | | | | | | | | | | |  |

Supplementary Table 7. Univariable and multivariable multinomial logistic regression model results for the association between ACEs and PLEs trajectory groups (20 imputations) for the sensitivity analysis restricted to participants with complete PLEs data (n= 1,761).

| **Characteristic** | | **Univariable Model** | | | | | | **Multivariable Model^** | | | | | | |
| --- | --- | --- | --- | --- | --- | --- | --- | --- | --- | --- | --- | --- | --- | --- |
|  |  | **Increasing PLEs** | | | **High PLEs** | | | **Increasing PLEs** | | | **High PLEs** | | | |
|  |  | Relative Risk Ratio | 95 % CI | | Relative Risk  Ratio | 95 % CI | | Relative Risk Ratio | 95 % CI | | Relative Risk Ratio | 95 % CI | |  |
|  |  |  | Low | High |  | High | Low |  | Low | High |  | Low | High |  |
| Maltreatment | | 2.00** | 1.31 | 3.06 | 2.08* | 1.17 | 3.68 | 1.87* | 1.14 | 3.06 | 2.29* | 1.16 | 4.52 |  |
| Bullying | | 2.39** | 1.47 | 3.89 | 1.95 | 0.97 | 3.93 | 2.35** | 1.39 | 3.96 | 1.84 | 0.86 | 3.93 |  |
| Intimate partner violence | | 1.44 | 0.89 | 2.32 | 1.44 | 0.75 | 2.76 | 1.09 | 0.61 | 1.96 | 1.11 | 0.50 | 2.46 |  |
| Parental mental health problems | | 1.46 | 0.95 | 2.25 | 1.21 | 0.66 | 2.22 | 1.20 | 0.72 | 2.00 | 0.96 | 0.45 | 2.02 |  |
| Parental drug abuse | | 1.12 | 0.61 | 2.04 | 0.55 | 0.20 | 1.52 | 0.83 | 0.43 | 1.63 | 0.38 | 0.13 | 1.14 |  |
| Parental separation | | 1.50 | 0.96 | 2.34 | 1.67 | 0.92 | 3.03 | 1.13 | 0.66 | 1.94 | 1.33 | 0.65 | 2.70 |  |
| Birth sex | |  |  |  |  |  |  |  |  |  |  |  |  |  |
|  | Male (ref) | 1 |  |  | 1 |  |  | 1 |  |  | 1 |  |  |  |
|  | Female | 0.75 | 0.51 | 1.10 | 1.21 | 0.70 | 2.10 | 0.80 | 0.53 | 1.19 | 1.03 | 0.57 | 1.85 |  |
| Low birthweight | | 0.44 | 0.11 | 1.80 | 0.88 | 0.21 | 3.70 |  | 0.34 | 0.08 | 1.47 | 0.77 | 0.77 |  |
| IQ | |  |  |  |  |  |  |  |  |  |  |  |  |  |
|  | Below average | 0.91 | 0.57 | 1.43 | 1.37 | 0.77 | 2.44 | 0.61 | 0.36 | 1.03 | 1.05 | 0.55 | 2.03 |  |
|  | Average or above (ref) | 1 |  |  | 1 |  |  | 1 |  |  | 1 |  |  |  |
| Paternal age | | 1.01 | 0.98 | 1.05 | 0.97 | 0.92 | 1.02 | 1.02 | 1.02 | 0.98 | 1.06 | 0.99 | 0.94 |  |
| Mother’s highest qualification | |  |  |  |  |  |  |  |  |  |  |  |  |  |
|  | CSE | 1.63 | 0.78 | 3.41 | 1.72 | 0.52 | 5.68 | 1.27 | 0.48 | 3.41 | 1.32 | 0.28 | 6.19 |  |
|  | Vocational | 2.76* | 1.36 | 5.63 | 2.46 | 0.74 | 8.19 | 2.33 | 0.95 | 5.71 | 2.29 | 0.53 | 9.82 |  |
|  | O-Level | 1.19 | 0.71 | 1.99 | 2.62* | 1.23 | 5.59 | 1.00 | 0.49 | 2.05 | 2.66 | 0.95 | 7.43 |  |
|  | A-Level | 0.86 | 0.49 | 1.50 | 1.20 | 0.51 | 2.82 | 0.78 | 0.41 | 1.49 | 1.18 | 0.44 | 3.17 |  |
|  | Degree or above (ref) | 1 |  |  | 1 |  |  | 1 |  |  | 1 |  |  |  |
| Mother’s partner’s highest qualification | |  |  |  |  |  |  |  |  |  |  |  |  |  |
|  | CSE | 1.44 | 0.79 | 2.63 | 1.44 | 0.59 | 3.54 | 0.88 | 0.39 | 1.96 | 0.67 | 0.20 | 2.17 |  |
|  | Vocational | 1.89 | 0.96 | 3.71 | 1.38 | 0.45 | 4.25 | 1.46 | 0.64 | 3.35 | 0.63 | 0.17 | 2.35 |  |
|  | O-Level | 0.70 | 0.38 | 1.29 | 1.42 | 0.68 | 2.97 | 0.56 | 0.27 | 1.17 | 0.82 | 0.32 | 2.07 |  |
|  | A-Level | 1.06 | 0.65 | 1.73 | 1.19 | 0.59 | 2.42 | 0.93 | 0.51 | 1.69 | 0.82 | 0.35 | 1.93 |  |
|  | Degree or above (ref) | 1 |  |  | 1 |  |  | 1 |  |  | 1 |  |  |  |
| Family income per week (£) | |  |  |  |  |  |  |  |  |  |  |  |  |  |
|  | <100 | 2.35 | 0.88 | 6.29 | 0.90 | 0.12 | 7.09 | 0.51 | 0.05 | 5.12 | 0.51 | 0.05 | 5.12 |  |
|  | 100 - 199 | 1.61 | 0.86 | 3.00 | 1.58 | 0.66 | 3.82 | 0.96 | 0.30 | 3.06 | 0.96 | 0.30 | 3.06 |  |
|  | 200 - 299 | 1.21 | 0.72 | 2.05 | 1.43 | 0.68 | 3.03 | 0.99 | 0.37 | 2.61 | 0.99 | 0.37 | 2.61 |  |
|  | 300- 399 | 0.95 | 0.53 | 1.68 | 1.12 | 0.52 | 2.44 | 0.84 | 0.35 | 2.02 | 0.84 | 0.35 | 2.02 |  |
|  | >400 (ref) | 1 |  |  | 1 |  |  | 1 |  |  | 1 |  |  |  |
| Mother’s social class | |  |  |  |  |  |  |  |  |  |  |  |  |  |
|  | I (ref) | 1 |  |  | 1 |  |  | 1 |  |  | 1 |  |  |  |
|  | II | 1.18 | 0.51 | 2.71 | 1.35 | 0.39 | 4.64 | 1.13 | 0.29 | 4.38 | 1.13 | 0.29 | 4.38 |  |
|  | III (non-manual) | 1.91 | 0.85 | 4.27 | 2.36 | 0.67 | 7.61 | 1.48 | 0.34 | 6.39 | 1.48 | 0.34 | 6.39 |  |
|  | III (manual) | 2.35 | 0.80 | 6.85 | 1.84 | 0.29 | 11.66 | 0.97 | 0.11 | 8.41 | 0.97 | 0.11 | 8.41 |  |
|  | IV | 1.60 | 0.53 | 4.80 | 4.19* | 1.06 | 16.54 | 2.83 | 0.52 | 15.29 | 2.83 | 0.52 | 15.29 |  |
|  | V | 2.40 | 0.46 | 12.60 | 2.79 | 0.27 | 27.98 | 1.67 | 0.12 | 22.98 | 1.67 | 0.12 | 22.98 |  |
| Does the mother own her own home (mortgaged or outright)? | |  |  |  |  |  |  |  |  |  |  |  |  |  |
|  | No | 1.31 | 0.73 | 2.35 | 1.56 | 0.73 | 3.35 | 0.94 | 0.47 | 1.88 | 1.25 | 0.49 | 3.17 |  |
|  | Yes (ref) | 1 |  |  | 1 |  |  | 1 |  |  | 1 |  |  |  |
| Neighbourhood deprivation quintile at child's birth (1: low deprived, 5: high deprived) | |  |  |  |  |  |  |  |  |  |  |  |  |  |
|  | 1 (ref) | 1 |  |  | 1 |  |  | 1 |  |  | 1 |  |  |  |
|  | 2 | 1.09 | 0.56 | 2.10 | 0.71 | 0.31 | 1.65 | 1.02 | 0.51 | 2.05 | 0.68 | 0.28 | 1.68 |  |
|  | 3 | 1.64 | 0.90 | 2.98 | 1.15 | 0.56 | 2.38 | 1.56 | 0.81 | 2.98 | 1.08 | 0.49 | 2.38 |  |
|  | 4 | 1.69 | 0.88 | 3.24 | 0.82 | 0.33 | 2.06 | 1.59 | 0.78 | 3.24 | 0.66 | 0.25 | 1.76 |  |
|  | 5 | 1.20 | 0.57 | 2.55 | 1.11 | 0.46 | 2.68 | 0.92 | 0.39 | 2.18 | 0.79 | 0.28 | 2.26 |  |
| Population density quintile at child's birth (inhab/km^2^), | |  |  |  |  |  |  |  |  |  |  |  |  |  |
|  | <=1000 (ref) | 1 |  |  | 1 |  |  | 1 |  |  | 1 |  |  |  |
|  | 2000 | 1.22 | 0.54 | 2.73 | 1.04 | 0.34 | 3.15 | 1.20 | 0.52 | 2.81 | 1.08 | 0.33 | 3.55 |  |
|  | 3000 | 1.02 | 0.42 | 2.52 | 0.59 | 0.14 | 2.49 | 1.07 | 0.41 | 2.79 | 0.60 | 0.13 | 2.77 |  |
|  | 4000 | 1.46 | 0.68 | 3.14 | 0.95 | 0.34 | 2.69 | 1.48 | 0.66 | 3.28 | 1.07 | 0.35 | 3.29 |  |
|  | 5000 | 1.06 | 0.44 | 2.54 | 1.10 | 0.36 | 3.37 | 1.11 | 0.44 | 2.78 | 1.20 | 0.35 | 4.11 |  |
|  | 6000 | 0.74 | 0.24 | 2.35 | 1.12 | 0.30 | 4.10 | 0.70 | 0.21 | 2.32 | 0.98 | 0.23 | 4.06 |  |
|  | 7000 | 1.13 | 0.40 | 3.18 | 0.97 | 0.24 | 3.99 | 1.02 | 0.34 | 3.01 | 1.11 | 0.24 | 5.00 |  |
|  | 8000 | 1.09 | 0.42 | 2.87 | 1.32 | 0.40 | 4.40 | 1.04 | 0.38 | 2.86 | 1.60 | 0.44 | 5.80 |  |
|  | 9000 | 0.72 | 0.19 | 2.73 | 1.10 | 0.26 | 4.66 | 0.73 | 0.18 | 2.92 | 1.41 | 0.30 | 6.56 |  |
|  | >=10000 | 1.27 | 0.50 | 3.22 | 1.02 | 0.28 | 3.68 | 1.14 | 0.43 | 3.07 | 1.24 | 0.31 | 4.94 |  |
| Mother or father depressed during pregnancy | | 1.30 | 1.30 | 0.80 | 2.12 | 1.33 | 0.68 | 0.94 | 0.53 | 1.67 | 0.94 | 0.41 | 2.16 |  |
| PRS for depression | | 1.01 | 1.01 | 0.83 | 1.22 | 1.26 | 0.96 | 1.00 | 0.82 | 1.23 | 1.26 | 0.95 | 1.67 |  |
| PRS for schizophrenia | | 1.07 | 1.07 | 0.88 | 1.30 | 1.06 | 0.81 | 1.04 | 0.85 | 1.28 | 0.99 | 0.75 | 1.31 |  |
| Principal component 1 | | 145 | 2.59e^-10^ | 8.13e^13^ | 1.09e^-10^ | 1.90e^-27^ | 6279141 | 4.29 | 1.30e^-12^ | 1.42e^13^ | 3.27e^-15^ | 5.13e^-33^ | 2090 |  |
| Principal component 2 | | 0.00031 | 1.30e^-11^ | 7173 | 3.53e^-07^ | 4.92e^-17^ | 2529 | 0.0024 | 5.10e^-11^ | 117516 | 8.07e^-06^ | 2.27e^-16^ | 286560 |  |
| Principal component 3 | | 0.014 | 7.06e^-10^ | 278143 | 121 | 9.68e^-09^ | 1.50e^12^ | 0.006 | 1.32e^-10^ | 313799 | 138 | 3.33e^-09^ | 5.74e^12^ |  |
| Principal component 4 | | 818 | 0.00003 | 2.64e^10^ | 4.81e^-07^ | 3.69e^-17^ | 6258 | 8943 | 0.00013 | 6.35e^11^ | 4.27e^-08^ | 1.08e^-18^ | 1682 |  |
| Principal component 5 | | 0.0035 | 1.52e^-10^ | 79541 | 3371667 | 0.00026 | 4.37e^16^ | 0.0013 | 1.72e^-11^ | 92103 | 4.38e^07^ | 0.0011 | 1.77e^18^ |  |
| Principal component 6 | | 14 | 4.96e^-07^ | 4.06e^08^ | 1.11e^07^ | 0.00063 | 1.95e^17^ | 10.53 | 1.85e^-07^ | 5.99e^08^ | 1884563 | 0.00005 | 7.08e^16^ |  |
| Principal component 7 | | 2529665 | 0.17 | 3.84e^13^ | 939 | 1.18e^-07^ | 7.46e^12^ | 2.03e^07^ | 0.43 | 9.53e^14^ | 86.19 | 3.23e^-09^ | 2.30e^12^ |  |
| Principal component 8 | | 299 | 0.00002 | 4.22e^09^ | 176 | 2.70e^-08^ | 1.14e^12^ | 350 | 0.00002 | 8.44e^+09^ | 77.5 | 1.76e^-09^ | 3.42e^12^ |  |
| Principal component 9 | | 6810 | 0.00040 | 1.15e^11^ | 1.51e^15^ | 209516 | 1.08e^25^ | 18858 | 0.0005 | 7.25e^11^ | 6.04e^15^ | 331676 | 1.10e^26^ |  |
| Principal component 10 | | 0.0018 | 8.40e^-11^ | 37990 | 0.36 | 3.20e^-12^ | 4.05e^08^ | 0.032 | 6.61e^-10^ | 1504108 | 0.02 | 4.66e^-13^ | 8.87e^08^ |  |
| * Indicates p<.05. ** indicates p<.001  *^ Adjusted for all variables in the model, including all covariates and other forms of ACEs.* | | | | | | | | | | | | | |  |

# References

Boyd, A., Golding, J., Macleod, J., Lawlor, D. A., Fraser, A., Henderson, J., Molloy, L., Ness, A., Ring, S., & Davey Smith, G. (2013). Cohort Profile: The ‘Children of the 90s’—the index offspring of the Avon Longitudinal Study of Parents and Children. *International Journal of Epidemiology*, *42*(1), 111–127. https://doi.org/10.1093/ije/dys064

Choi, S. W., & O’Reilly, P. F. (2019). PRSice-2: Polygenic Risk Score software for biobank-scale data. *GigaScience*, *8*(7), giz082. https://doi.org/10.1093/gigascience/giz082

Diop, A., Gupta, A., Mueller, S., Dron, L., Harari, O., Berringer, H., Kalatharan, V., Park, J. J. H., Mésidor, M., & Talbot, D. (2024). Assessing the performance of group-based trajectory modeling method to discover different patterns of medication adherence. *Pharmaceutical Statistics*, *23*(4), 511–529. https://doi.org/10.1002/pst.2365

Fisher, H. L., Schreier, A., Zammit, S., Maughan, B., Munafò, M. R., Lewis, G., & Wolke, D. (2013). Pathways between childhood victimization and psychosis-like symptoms in the ALSPAC birth cohort. *Schizophrenia Bulletin*, *39*(5), 1045–1055. https://doi.org/10.1093/schbul/sbs088

Gill, B. (2015). *The English Indices of Deprivation 2015*. Department for Communities and Local Government.

Herle, M., Micali, N., Abdulkadir, M., Loos, R., Bryant-Waugh, R., Hübel, C., Bulik, C. M., & De Stavola, B. L. (2020). Identifying typical trajectories in longitudinal data: Modelling strategies and interpretations. *European Journal of Epidemiology*, *35*(3), 205–222. https://doi.org/10.1007/s10654-020-00615-6

Horwood, J., Salvi, G., Thomas, K., Duffy, L., Gunnell, D., Hollis, C., Lewis, G., Menezes, P., Thompson, A., Wolke, D., Zammit, S., & Harrison, G. (2008). IQ and non-clinical psychotic symptoms in 12-year-olds: Results from the ALSPAC birth cohort. *The British Journal of Psychiatry*, *193*(3), 185–191. https://doi.org/10.1192/bjp.bp.108.051904

Jones, B. L., & Nagin, D. S. (2013). A Note on a Stata Plugin for Estimating Group-based Trajectory Models. *Sociological Methods & Research*, *42*(4), 608–613. https://doi.org/10.1177/0049124113503141

Kwong, A. S. F., Morris, T. T., Pearson, R. M., Timpson, N. J., Rice, F., Stergiakouli, E., & Tilling, K. (2021). Polygenic risk for depression, anxiety and neuroticism are associated with the severity and rate of change in depressive symptoms across adolescence. *Journal of Child Psychology and Psychiatry*, *62*(12), 1462–1474. https://doi.org/10.1111/jcpp.13422

Levis, B., Negeri, Z., Sun, Y., Benedetti, A., & Thombs, B. D. (2020). Accuracy of the Edinburgh Postnatal Depression Scale (EPDS) for screening to detect major depression among pregnant and postpartum women: Systematic review and meta-analysis of individual participant data. *BMJ*, *371*, m4022. https://doi.org/10.1136/bmj.m4022

Logeswaran, Y., Dykxhoorn, J., Dalman, C., & Kirkbride, J. B. (2023). Social Deprivation and Population Density Trajectories Before and After Psychotic Disorder Diagnosis. *JAMA Psychiatry*, *80*(12), 1258–1268. https://doi.org/10.1001/jamapsychiatry.2023.3220

Martin, A. R., Kanai, M., Kamatani, Y., Okada, Y., Neale, B. M., & Daly, M. J. (2019). Clinical use of current polygenic risk scores may exacerbate health disparities. *Nature Genetics*, *51*(4), 584–591. https://doi.org/10.1038/s41588-019-0379-x

Nagin, D. S., & Odgers, C. L. (2010). Group-Based Trajectory Modeling in Clinical Research. *Annual Review of Clinical Psychology*, *6*(1), 109–138. https://doi.org/10.1146/annurev.clinpsy.121208.131413

Nylund-Gibson, K., & Choi, A. Y. (2018). Ten frequently asked questions about latent class analysis. *Translational Issues in Psychological Science*, *4*(4), 440–461. https://doi.org/10.1037/tps0000176

Raftery, A. (1995). Bayesian Model Selection in Social Research. *Sociological Methodology*, *25*, 111–163.

*Standard Occupational Classification*. (1991). Her Majesty’s Stationery Office. https://ukdataservice.ac.uk/app/uploads/1991_defs.pdf

The Schizophrenia Working Group of the Psychiatric Genomics Consortium, Ripke, S., Walters, J. T., & O’Donovan, M. C. (2020). *Mapping genomic loci prioritises genes and implicates synaptic biology in schizophrenia* (p. 2020.09.12.20192922). medRxiv. https://doi.org/10.1101/2020.09.12.20192922

Trubetskoy, V., Pardiñas, A. F., Qi, T., Panagiotaropoulou, G., Awasthi, S., Bigdeli, T. B., Bryois, J., Chen, C.-Y., Dennison, C. A., Hall, L. S., Lam, M., Watanabe, K., Frei, O., Ge, T., Harwood, J. C., Koopmans, F., Magnusson, S., Richards, A. L., Sidorenko, J., … O’Donovan, M. C. (2022). Mapping genomic loci implicates genes and synaptic biology in schizophrenia. *Nature*, *604*(7906), 502–508. https://doi.org/10.1038/s41586-022-04434-5

*UNICEF-WHO Joint Database on Low birth weight*. (2023). https://data.unicef.org/topic/nutrition/low-birthweight/#data

Wechsler, D., Golombok, S., & Rust, J. (1992). Weschler Intelligence Scale for Children (3rd Edition) (WISC–III UK). *The Psychological Corporation*.

Wray, N. R., Ripke, S., Mattheisen, M., Trzaskowski, M., Byrne, E. M., Abdellaoui, A., Adams, M. J., Agerbo, E., Air, T. M., Andlauer, T. M. F., Bacanu, S.-A., Bækvad-Hansen, M., Beekman, A. F. T., Bigdeli, T. B., Binder, E. B., Blackwood, D. R. H., Bryois, J., Buttenschøn, H. N., Bybjerg-Grauholm, J., … Sullivan, P. F. (2018). Genome-wide association analyses identify 44 risk variants and refine the genetic architecture of major depression. *Nature Genetics*, *50*(5), 668–681. https://doi.org/10.1038/s41588-018-0090-3
